# Supramolecular Chiral Binding Affinity‐Achieved Efficient Synergistic Cancer Therapy

**DOI:** 10.1002/advs.202308493

**Published:** 2024-02-21

**Authors:** Jinfeng Zhou, Jiake Gu, Xiaohuan Sun, Qianyun Ye, Xuan Wu, Juqun Xi, Jie Han, Yu Liu

**Affiliations:** ^1^ School of Chemistry and Chemical Engineering Yangzhou University Yangzhou Jiangsu 225002 P. R. China; ^2^ School of Medicine Yangzhou University Yangzhou Jiangsu 225002 P. R. China; ^3^ College of Chemistry State Key Laboratory of Elemento‐Organic Chemistry Nankai University Tianjin 300071 P. R. China

**Keywords:** chirality transfer, chirality‐specific cellular uptake, photothermal therapy, supramolecular chirality

## Abstract

Supramolecular chirality‐mediated selective interaction among native assemblies is essential for precise disease diagnosis and treatment. Herein, to fully understand the supramolecular chiral binding affinity‐achieved therapeutic efficiency, supramolecular chiral nanoparticles (WP5⊃D/L‐Arg+DOX+ICG) with the chirality transfer from chiral arginine (D/L‐Arg) to water‐soluble pillar[5]arene (WP5) are developed through non‐covalent interactions, in which an anticancer drug (DOX, doxorubicin hydrochloride) and a photothermal agent (ICG, indocyanine green) are successfully loaded. Interestingly, the WP5⊃D‐Arg nanoparticles show 107 folds stronger binding capability toward phospholipid‐composed liposomes compared with WP5⊃L‐Arg. The enantioselective interaction further triggers the supramolecular chirality‐specific drug accumulation in cancer cells. As a consequence, WP5⊃D‐Arg+DOX+ICG exhibits extremely enhanced chemo‐photothermal synergistic therapeutic efficacy (tumor inhibition rate of 99.4%) than that of WP5⊃L‐Arg+DOX+ICG (tumor inhibition rate of 56.4%) under the same condition. This work reveals the breakthrough that supramolecular chiral assemblies can induce surprisingly large difference in cancer therapy, providing strong support for the significance of supramolecular chirality in bio‐application.

## Graphical abstract

Supramolecular chiral nanomaterials with the chirality transfer from D/L‐Arg to WP5 are constructed. In contrast with WP5⊃L‐Arg+DOX+ICG, supramolecular nanoparticles in D‐form show enhanced cellular uptake efficiency and improved chemo‐photothermal synergistic therapy.

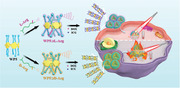

## 1 Introduction

Supramolecular assembly has gained increasing attention in the field of bioapplication, specifically in biosensing,^[^ ^1^ ^]^ cellular uptake,^[^ ^2^ ^]^ in situ self‐assembly,^[^ ^3^ ^]^ and drug delivery.^[^ ^4^ ^]^ Among the various reports, macrocyclic host, particularly pillar[n]arene‐mediated supramolecular nanomaterials have shown promising therapeutic efficacy.^[^ ^5^, ^6^, ^7^, ^8^ ^]^ Along with research line, many pillar[n]arene‐based stimuli‐responsive therapeutic systems have been successfully constructed, in which the tumor‐targeting delivery systems,^[^ ^9^, ^10^ ^]^ as well as the combined therapeutic systems^[^ ^11^, ^12^ ^]^ could be simply achieved by the encapsulation of various guest molecules into pillar[n]arenes. Although continuous advancements have been achieved, the exploration of supramolecular chiral assemblies remains limited in this domain. As is well known, there exists extensive interaction among the proteins or other native assemblies in vivo, which play vital roles not only in physicological processes, such as genetic information transfer and storage, cellular metabolism, but also in disease biology,^[^ ^13^ ^]^ proteomics,^[^ ^14^ ^]^ and neurobiology.^[^ ^15^ ^]^ Among all the factors afftecting these processes, the chirality from these assemblies, reffered as supramolecular chirality, is one of the key points. For example, the right‐handed double helix structure of DNA is crucial to DNA transcript, translation, and gene expression, while the secondary structure of protein plays a vital role in maintaining structural stability and enzymatic activity. Therefore, the fully understanding of supramolecular chirality is essential for comprehending the intricate mechanisms underlying these fundamental biological processes and will provide revolutionary insights for biotherapy.

Supramolecular chirality, resulting from the symmetry breaking during the self‐assembly processes,^[^ ^16^ ^]^ surpasses molecular chirality in several aspects.^[^ ^17^ ^]^ First of all, the self‐assembly building blocks could be flexibly designed to construct various chiral nanostructures with specific function,^[^ ^18^, ^19^ ^]^ enabling them excellent candidates for various fields. In addition, due to the dynamic nature of self‐assembly, the chiral properties could be finely tuned by external stimuli, such as light,^[^ ^20^ ^]^ solvents,^[^ ^21^ ^]^ temperature,^[^ ^22^ ^]^ etc. Moreover, supramolecular chiral assemblies often create a multivalent binding environment,^[^ ^23^ ^]^ which can enhance the overall affinity and enantioselectivity to chiral target species. Last but not least, the “sergeants‐and‐soldiers” and the “majority‐rules” principles play vital roles in the self‐assembly processes, leading to chirality transfer and chirality amplification, which will further facilitate the enhancement in enantioselective capability and improvement in the fields of chiral catalysis, chiral sensing, chiroptic materials, etc.^[^ ^24^, ^25^ ^]^ Taken together, the design and manipulation of supramolecular chirality is of great significance. In particular, in the therapeutic systems, the introduction of supramolecular chirality may realize high drug utilization efficiency, precise diagnosis, and treatments. Though the differential biologiacl effect induced by molecular chirality was reproted,^[^ ^26^ ^]^ the potential function of supramolecular chirality in this aspect is yet to be fully understood.

**Scheme 1:**
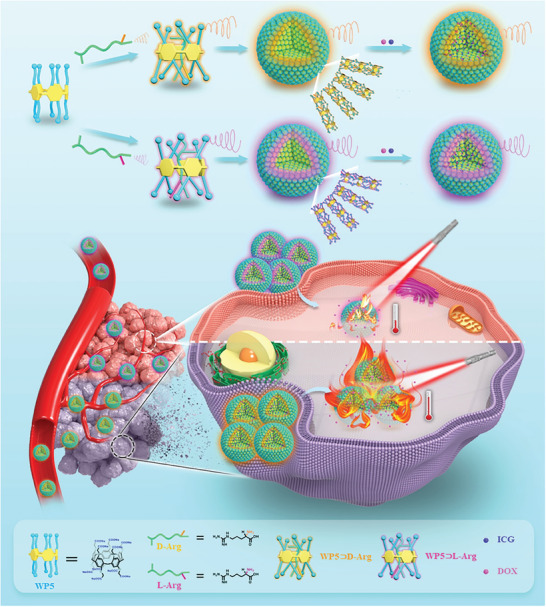
Schematic representation of the construction of chiral WP5⊃D/L‐Arg+DOX+ICG supramolecular nanoparticles and corresponding chirality‐specific chemo‐photothermal synergistic therapy.

Herein, supramolecular chiral assemblies (WP5⊃D/L‐Arg) have been successfully constructed with water‐soluble pillar[5]arene (WP5) and chiral arginine (D/L‐Arg) (**Scheme** **1**). The chirality transfer from D/L‐Arg to WP5 was demonstrated for WP5⊃D/L‐Arg. When incubated with chiral liposomes or cells, WP5⊃D‐Arg showed much higher internalization efficiency than WP5⊃L‐Arg due to chirality‐mediated binding behavior. On this basis, we encapsulated the anti‐cancer drug doxorubicin hydrochloride (DOX) and photothermal agent indocyanine green (ICG) into WP5⊃D/L‐Arg, in which the identical DOX/ICG encapsulation efficiency, drug release behavior and photothermal performance could be realized. However, due to the differential cellular uptake efficiency, supramolecular chirality‐specific cancer therapeutic efficacy was achieved, with the WP5⊃D‐Arg+DOX+ICG showing higher tumor inhibition efficiency than WP5⊃L‐Arg+DOX+ICG under NIR irradiation. This study discovers that supramolecular chirality can induce surprisingly large difference in cancer therapy and thus, can open up a brand‐new perspective in engineering high‐level biomaterials. Additionally, clarifying the role of supramolecular chirality in biotherapy will significantly enhance our understanding of the existence of supramolecular chiral motifs (e.g., DNA, proteins) within living systems.

## 2 Results and Discussion

### 2.1 Supramolecular Chiral Assemblies Engineering and Characterization

WP5, D‐Arg, DL‐Arg (racemic Arg) and L‐Arg were employed for the construction of supramolecular chiral assemblies herein. We chose WP5 and Arg for several reasons. First, WP5 is a typical host molecule with electron‐rich property, a hydrophobic cavity, and functional carboxylate groups that endow it a high affinity for chiral Arg.^[^ ^27^, ^28^ ^]^ Second, WP5 has planar chirality. Though usually exists in racemic form due to the fast rotation of the phenyl rings, the planar chirality of WP5 can be induced and stabilized by chiral guest molecules.^[^ ^29^ ^]^ Therefore, the chirality of Arg can be transferred to WP5 via host‐guest interaction, resulting in assemblies with amplified supramolecular chirality. Lastly, the “Arg magic”, i.e., the outstanding capability of arginine‐rich species for cellular uptake, has been well‐demonstrated,^[^ ^30^, ^31^ ^]^ which makes Arg an excellent candidate for engineering chiral supramolecular nanomaterials for deeper understanding of nanobio‐interaction.

**Figure 1:**
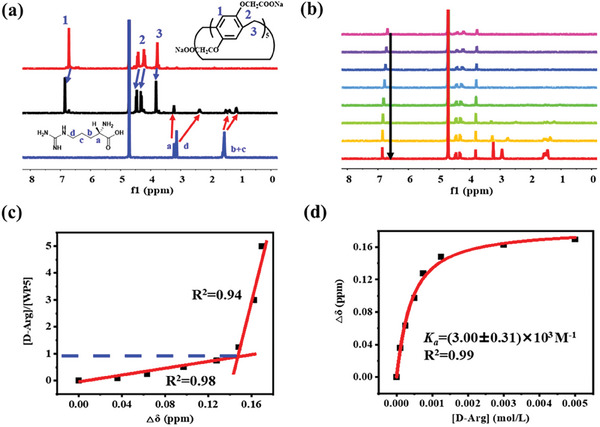
a) ^1^H NMR spectra (400 MHz, D_2_O, 298 K, pD = 7.2) of WP5, WP5⊃D‐Arg (molar ratio 1:1), and D‐Arg. b) ^1^H NMR spectra (400 MHz, D_2_O, 298 K, pD = 7.2) of WP5 at a concentration of 1.0 mm upon addition of D‐Arg. From top to bottom, the concentration of D‐Arg was kept at 0, 0.1, 0.25, 0.5, 0.75, 1.25, 3.0, 5.0 mm. c) Plot of the molar ratio of [D‐Arg]/[WP5] against the Δδ of H_1_ of WP5. d) Plot of Δδ of H_1_ against the concentration of D‐Arg. The red line was obtained from non‐linear curve‐fitting.

The host‐guest interaction between WP5 and D/DL/L‐Arg was first investigated by proton nuclear magnetic resonance (^1^H NMR) spectroscopy. As revealed from **Figure** **1**a, when in the presence of WP5, the protons derived from the methylene protons of D‐Arg exhibited evident upfield shift and broadening in comparison with the individual D‐Arg as a consequence of inclusion induced shielding effects. For the α proton, the signal was slightly upshifted, indicating the chiral center of Arg is in close proximity to the cavity portal of WP5 and the amino group and carboxylate group were located outside the WP5 cavity. In addition, when mixed with D‐Arg, the signals arisen from the aromatic and methylene protons of WP5 underwent obvious downfield shifts. The same results were obtained in the case of DL‐Arg and L‐Arg as well (Figure S1, Supporting Information). All the above evidences suggested the host‐guest complexation between WP5 and D/DL/L‐Arg. To further study the stoichiometry of the WP5⊃D/DL/L‐Arg host‐guest complex, ^1^H NMR titration experiments were carried out. The ^1^H NMR spectra of WP5 with varying concentration of Arg were given in Figure 1b and Figure S2 (Supporting Information). From Figure 1b and Figure S2 (Supporting Information), it is clear that with the concentration increasing of Arg, the proton signals from both WP5 and Arg were shifted gradually. By plotting the molar ratio of [D‐Arg]/[WP5] against the Δδ of H_1_ of WP5, a turning point of Δδ was observed at the 1:1 molar ratio of WP5: D/L‐Arg (Figure 1c; Figure S3, Supporting Information), which clearly confirmed the 1:1 binding mode between WP5 and Arg. Furthermore, the apparent association constants (*K*_a_) between WP5 and D/DL/L‐Arg were further calculated to be (3.00 ± 0.31) × 10^3^ m ^−1^, (3.10 ± 0.48) × 10^3^ m ^−1^, (3.03 ± 0.36) × 10^3^ m ^−1^, respectively, by plotting the Δδ of H_1_ of WP5 against the concentration of Arg (Figure 1d; Figure S4, Supporting Information). The above results indicated the relative strong and chirality‐independent binding affinity between Arg and WP5.

**Figure 2:**
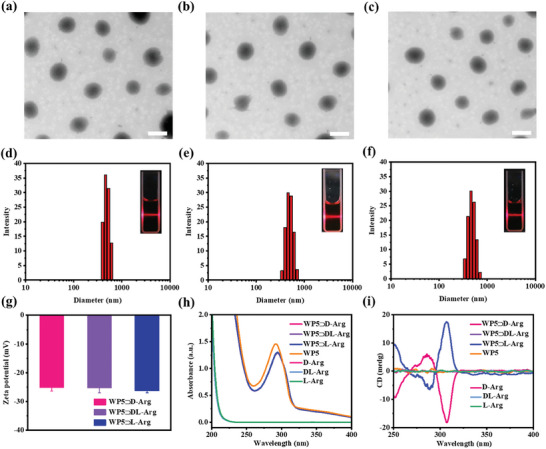
TEM images of a) WP5⊃D‐Arg, b) WP5⊃DL‐Arg, and c) WP5⊃L‐Arg in aqueous solution. Scale bar: 500 nm. DLS results of d) WP5⊃D‐Arg, e) WP5⊃DL‐Arg, and f) WP5⊃L‐Arg in aqueous solution. The insets show the Tyndall effect of corresponding mixture. g) Zeta potential of WP5⊃D/DL/L‐Arg in aqueous solution. h) UV–vis spectra and i) CD spectra of WP5⊃D/DL/L‐Arg, WP5 and D/DL/L‐Arg in aqueous solution.

With the careful observation of the structure of WP5⊃D/DL/L‐Arg, we assume the partial protonation of WP5 and the positive charged guanidium group of Arg may induce intermolecular hydrogen bonding and electrostatic interactions and, in further, the assembly of WP5⊃D/DL/L‐Arg. Therefore, the morphology of WP5⊃D/DL/L‐Arg was subsequently studied by TEM. As revealed from **Figure** **2**a–c, spherical nanoparticles with an average diameter of ≈300 nm were observed for all the three cases. With careful observation, the supramolecular nanoparticles obtained were featured with core‐shell characteristic. From Figure S5 (Supporting Information), the core and shell components can be easily differentiated by brightness difference. The formation of core‐shell nanostructure can be attributed to the multi‐micelle aggregation mechanism induced phase separation as we previously reported.^[^ ^32^ ^]^ In addition, from the dynamic light scattering (DLS) results, the average hydrated diameters of the WP5⊃D/DL/L‐Arg supramolecular nanostructure were determined to be ≈400 nm, which is in consistent with the TEM results. The Tyndall effect showed in the inset of Figure 2d–f further proved the assembly of WP5⊃D/DL/L‐Arg. Besides, according to Figure 2g, the zeta potential of WP5⊃D/DL/L‐Arg nanoparticles reached ≈‐25 mV, indicating their excellent colloidal stability.

The UV–vis absorption spectra of the WP5⊃D/DL/L‐Arg supramolecular nanoparticles were subsequently recorded. As depicted in Figure 2h, the presence of D/DL/L‐Arg resulted in a shift of the maximum absorption wavelength of WP5 from 290 to 295 nm, providing additional evidence for the assembly of WP5⊃D/DL/L‐Arg. When looking at the CD spectra of WP5, D/DL/L‐Arg, and WP5⊃D/DL/L‐Arg (Figure 2i), it is obvious that no evident signals were observed for WP5, D/DL/L‐Arg, and WP5⊃DL‐Arg in the range of 200–450 nm. However, the WP5⊃D‐Arg showed a clear exciton‐type CD spectrum, with a strong positive Cotton effect at ≈290 nm and a negative Cotton effect at ≈310 nm. Under the same condition, WP5⊃L‐Arg exhibited completely reversed CD signals, indicating the D‐Arg and L‐Arg can induce opposite chirality of corresponding supramolecular nanoparticles. Upon further inspection, the crossover of the exciton CD spectrum was located at 295 nm, which exactly overlapped with the maximum UV–vis absorption of WP5 from WP5⊃D/L‐Arg assemblies. The results clearly indicated that the newly emerging CD peaks were derived from WP5. Taken together, the above data provides clear confirmation that chirality transfer from chiral Arg to WP5 occurred and as a consequence, chirality exists throughout the resultant supramolecular nanoparticles.

### 2.2 Chirality‐Dependent Interaction and Cellular Uptake

**Figure 3:**
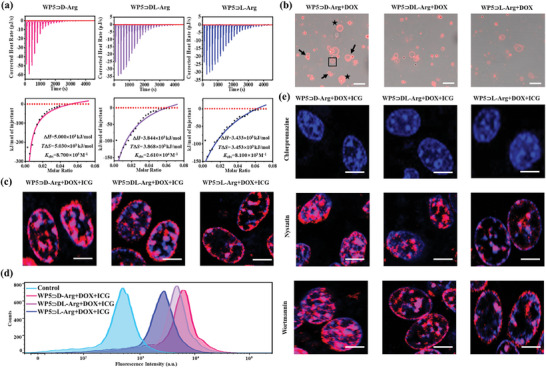
a) ITC results of the addition of WP5⊃D‐Arg, WP5⊃DL‐Arg and WP5⊃L‐Arg into liposome dispersion. b) Confocal fluorescence images of the mixture of WP5⊃D/DL/L‐Arg+DOX (red) and liposomes. Scale bars: 5 µm. c) Cellular uptake of WP5⊃D/DL/L‐Arg+DOX+ICG (red) into DAPI‐stained CT26 cell nuclei (blue) at 37 °C. Scale bars: 5 µm. d) Flow cytometry data showing the fluorescence intensity of DOX in WP5⊃D/DL/L‐Arg+DOX+ICG treated CT26 cells. e) Cellular uptake of WP5⊃D/DL/L‐Arg+DOX+ICG (red) into DAPI‐stained CT26 cell nuclei (blue) in the presence of various endocytosis inhibitors. Scale bars: 5 µm.

To verify the possible chirality‐specific bio‐application of WP5⊃D/DL/L‐Arg, liposomes, which mimic the cell membrane, formed by chiral soy lecithin were prepared with a typical approach.^[^ ^33^ ^]^ As revealed from the confocal microscopic image (Figure S6, Supporting Information), the liposomes with spherical shape were well distributed and their diameters were ≈1 µm. Subsequently, to gain a quantitative understanding of the binding affinity between WP5⊃D/DL/L‐Arg and liposomes, the WP5⊃D/DL/L‐Arg nanoparticles were separately added into a liposome dispersion and the isothermal titration calorimetry (ITC) was measured, respectively. As shown in **Figure** **3**a, with the addition of WP5⊃D‐Arg, the enthalpy change obtained was more significant than that of WP5⊃DL‐Arg and WP5⊃L‐Arg. The *K*_obs_ between WP5⊃D‐Arg and liposomes was determined to be much higher than that of WP5⊃DL‐Arg or WP5⊃L‐Arg. Specifically, the *K*_obs_ of WP5⊃DL‐Arg or WP5⊃L‐Arg toward liposomes was calculated to be 2.610 × 10^4^ m ^−1^ or 8.100 × 10^3^ m ^−1^, which is ≈33 or 107 folds lower in comparison with that of WP5⊃D‐Arg (8.700 × 10^5^ m ^−1^). Moreover, according to the thermodynamic parameters, a more negative value of Δ*G* was obtained in the case of WP5⊃D‐Arg, further confirming the energetically favorable binding between WP5⊃D‐Arg and liposomes. To prove the universality of the above phenomenon, WP5⊃D/L‐lysine (Lys) and WP5⊃D/L‐alanine‐arginine (Ala‐Arg) supramolecular assemblies with evident Tyndall effect were prepared (Figure S7, Supporting Information). Through the enhancement in UV–vis absorption induced by liposomes (Figure S8, Supporting Information), the stronger binding affinity of D‐formed nanoparticles toward liposomes were verified. From another point of view, when Arg molecule was investigated, we found that the *K*_obs_ between D‐Arg and liposome was limited to 2.300 × 10^3^ m ^−1^ (Figure S9, Supporting Information), which clearly illustrated the superior binding affinity of WP5⊃D/DL/L‐Arg supramolecular assemblies in the context of biological application.

As stronger nanoparticle‐liposome binding affinity can lead to higher internalization efficiency, we assume the differential binding capability of WP5⊃D/DL/L‐Arg supramolecular assemblies toward liposomes can lead to distinct internalization efficiency. To confirm the above assumption, the WP5⊃D/DL/L‐Arg supramolecular nanoparticles were loaded with fluorescent DOX. The successful encapsulation of DOX was verified by the emergence of UV–vis absorption peaks ≈490 nm (Figure S10, Supporting Information) and the detection of red fluorescence through confocal fluorescence microscopy (Figure S11, Supporting Information) of WP5⊃D/DL/L‐Arg+DOX. After the incubation of WP5⊃D/DL/L‐Arg+DOX (red fluorescence) with liposomes (no fluorescence) for 24 h, the resulting mixtures were subsequently measured using confocal fluorescence microscopy (Figure 3b). At first glance, it is of interest that most of the WP5⊃D‐Arg+DOX nanoparticles were located around the surface of the liposomes (indicated by the arrows). While in the other cases, a lower percent of WP5⊃DL‐Arg+DOX nanoparticles were bound with liposomes and the WP5⊃L‐Arg+DOX nanoparticles were mostly separated with liposomes, which suggests the strong binding affinity between WP5⊃D‐Arg+DOX and liposomes. Additionally, from Figure 3b, it is also evident that the bilayers of the liposomes were featured with red fluorescence (indicated by the square frame). We assume that this was caused by the entrance of the small‐sized WP5⊃D/DL/L‐Arg+DOX nanoparticles. With the quantification of the fluorescent intensity of the bilayers, the highest fluorescence intensity was obtained from WP5⊃D‐Arg+DOX, followed with WP5⊃DL‐Arg+DOX and WP5⊃L‐Arg+DOX (Figure S12, Supporting Information). Moreover, in contrast with the cases of WP5⊃DL/L‐Arg+DOX nanoparticles, the internalization of WP5⊃D‐Arg+DOX nanoparticles into liposomes were clearly observed (indicated by the stars), which can be ascribed to the strongest binding affinity of WP5⊃D‐Arg+DOX to liposomes. Given that the liposomes are chiral and all the WP5⊃D/DL/L‐Arg+DOX nanoparticles have the identical molecular and supramolecular structure, the differentiated binding affinity and internalization efficiency can be only ascribed to supramolecular chirality‐specific interaction.

Inspired by the above positive results, the WP5⊃D/DL/L‐Arg supramolecular nanoparticles were further endowed with anti‐tumor capability. Specifically, WP5⊃D/DL/L‐Arg supramolecular nanoparticles were mixed with DOX (anti‐cancer drug) and ICG (photothermal agent) for 24 h. After dialyzing, the unbound free molecules were completely removed, resulting WP5⊃D/DL/L‐Arg+DOX+ICG. From the UV–vis spectra of WP5⊃D/DL/L‐Arg+DOX+ICG (Figure S13, Supporting Information), it is obvious that new peaks centered at ≈490 and ≈780 nm appeared. The above result confirmed the successful fabrication of WP5⊃D/DL/L‐Arg+DOX+ICG. According to the standard curves of DOX and ICG (Figure S14, Supporting Information), the encapsulation efficiency of DOX was further demonstrated to be 81.64%, 81.52%, and 81.76% and that of ICG was calculated to be 74.60%, 75.62% and 74.95 for WP5⊃D/DL/L‐Arg+DOX+ICG, respectively. Moreover, in comparison with the CD spectra of WP5⊃D/DL/L‐Arg, no obvious change of the CD signal of WP5 from WP5⊃D/DL/L‐Arg+DOX+ICG was observed (Figure S15, Supporting Information), indicating the similar chiral feature of WP5⊃D/DL/L‐Arg and WP5⊃D/DL/L‐Arg+DOX+ICG.

To further verify the chirality‐specific interactions in biological system, the resulting WP5⊃D/DL/L‐Arg+DOX+ICG supramolecular nanoparticles (red fluorescence) were incubated with DAPI (blue fluorescence)‐stained CT26 cells for 24 h followed with substantial rinses to remove the unbound nanoparticles. Subsequently, the confocal fluorescence microscopy observation was performed. From Figure 3c, we found that a noticeable higher amount of WP5⊃D‐Arg+DOX+ICG nanoparticles were internalized inside the CT26 cells compared to WP5⊃DL‐Arg+DOX+ICG and WP5⊃L‐Arg+DOX+ICG nanoparticles. Additionally, the CT26 cells with various treatments were analyzed by flow cytometry. In contrast with the control group, the WP5⊃D/DL/L‐Arg+DOX+ICG treated cells showed significant stronger fluorescence intensity, indicating the cellular internalizaiton of WP5⊃D/DL/L‐Arg+DOX+ICG nanoparticles. In addition, the mean fluorescence intensity of WP5⊃D/DL/L‐Arg+DOX+ICG treated cells was determined to be 7.1 × 10^3^, 5.6 × 10^3^, and 3.2 × 10^3^ via FITC channel (Figure 3d), confirming the ≈1.3 and ≈2.2 folds higher internalization amount of WP5⊃D‐Arg+DOX+ICG than WP5⊃DL‐Arg+DOX+ICG and WP5⊃L‐Arg+DOX+ICG. To further investigate the chirality‐specific internalization of WP5⊃D/L‐Arg+DOX+ICG supramolecular nanoparticles, the cellular uptake efficiency was separately investigated on HK‐2 cells (normal cells), RAW264.7 cells (normal cells) and B16 cells (cancer cells). From the confocal fluorescence images (Figure S16, Supporting Information), higher internalization efficiency of WP5⊃D‐Arg+DOX+ICG nanoparticles than WP5⊃L‐Arg+DOX+ICG was observed, indicating the universality of the chirality‐specific cellular internalization performance. These findings above confirmed the supramolecular chirality‐dependent cellular uptake of WP5⊃D/DL/L‐Arg+DOX+ICG and revealed the potential of supramolecular chirality‐induced bio‐applications.

The cellular uptake mechanism of WP5⊃D/DL/L‐Arg+DOX +ICG supramolecular nanoparticles into CT26 cell was then investigated. It is known direct penetration and endocytosis are the main pathways for the cellular uptake of nanomaterials.^[^ ^34^, ^35^ ^]^ To clarify if the cellular uptake of WP5⊃D/DL/L‐Arg+DOX+ICG supramolecular nanoparticles is mediated by direct penetration pathway (energy independent), the cellular uptake experiments were performed at 4 °C, with the co‐incubation of WP5⊃D/DL/L‐Arg+DOX+ICG nanoparticles and Hela cells for 24 h. As shown in Figure S17 (Supporting Information), the internalization of WP5⊃D/DL/L‐Arg+DOX+ICG into CT26 cells was completely inhibited at this condition, suggesting the endocytosis‐based cellular uptake of WP5⊃D/DL/L‐Arg+DOX+ICG. To further demonstrate the specific endocytosis pathway, inhibitors, i.e., chlorpromazine (inhibitor of clathrin‐dependent endocytosis), nystatin (inhibitor of caveolin‐dependent endocytosis), and wortmannin (inhibitor of micropinocytosis‐mediated endocytosis) were employed for the pretreatment of CT26 cells. From Figure 3e, it is clear that with the addition of nystatin and wortmannin, after 24 h co‐incubation, the cellular uptake efficiency of WP5⊃D/DL/L‐Arg+DOX+ICG did not change. However, when treated with chlorpromazine, the cellular uptake process of WP5⊃D/DL/L‐Arg+DOX+ICG was completely blocked. The above results confirmed that the cellular uptake of WP5⊃D/DL/L‐Arg+DOX+ICG into CT26 cells was triggered through clathrin‐dependent manner. It is known that the cellular uptake of nanomaterials includes their absorption on the cell membrane followed by internalization.^[^ ^36^ ^]^ Since both the cell membrane and clathrin receptor are chiral, the enantioselective interactions between them and WP5⊃D/DL/L‐Arg+DOX+ICG undoubtedly induced the supramolecular chirality‐dependent cellular uptake efficiency.

### 2.3 Photothermal Performance and Controlled Drug Release

**Figure 4:**
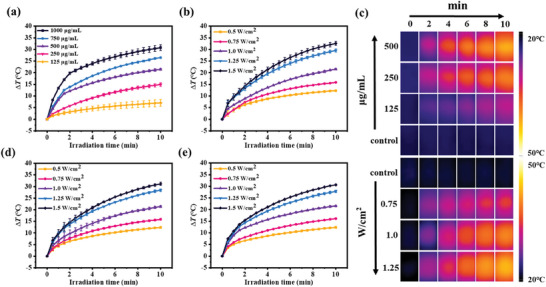
a) Photothermal heating curves of WP5⊃D‐Arg+DOX+ICG aqueous solution at different concentrations under 808 nm laser irradiation (10 min, 1.0 W cm^−2^). b) Photothermal heating curves of WP5⊃D‐Arg+DOX+ICG aqueous solution (500 µg mL^−1^) under the irradiation of 808 nm laser at various power densities for 10 min. c) Infrared thermal images of WP5⊃D‐Arg+DOX+ICG at different conditions. Photothermal curves of d) WP5⊃DL‐Arg+DOX+ICG aqueous solution and e) WP5⊃L‐Arg+DOX+ICG aqueous solution (500 µg mL^−1^) under the irradiation of 808 nm laser at various powder densities for 10 min. Data were presented as mean ± SD (*n* = 3).

Based on our previous discussion, it can be inferred that the successful encapsulation of ICG within WP5⊃D/DL/L‐Arg+DOX+ICG supramolecular nanoparticles has led to significant absorption within NIR‐I range. As a consequence, it is reasonable to assume that these supramolecular nanoparticles exhibit photothermal capabilities. Taking this in mind, the photothermal performance of WP5⊃D/DL/L‐Arg+DOX+ICG was investigated with an 808 nm laser. From the photothermal curves, it is clear that the photothermal performance of WP5⊃D‐Arg+DOX+ICG nanoparticles was positively correlated with the concentration, laser power density, and irradiation time (**Figure** **4**a,b). When the WP5⊃D‐Arg+DOX+ICG solution (500 µg mL^−1^) was exposed to 808 nm laser (1.0 W cm^−2^), the temperature of the solution increased ∼25 °C and reached ∼46 °C, which is considered to be desirable for cancer photothermal therapy. The photothermal performance of WP5⊃D‐Arg+DOX+ICG was also verified by the infrared thermal images (Figure 4c). What is also worth to mention, the photothermal behavior of WP5⊃DL‐Arg+DOX+ICG and WP5⊃L‐Arg+DOX+ICG is almost identical with that of WP5⊃D‐Arg+DOX+ICG due to the similar encapsulation efficiency of ICG (Figure 4d,e). Furthermore, to demonstrate the photothermal stability of the WP5⊃D/DL/L‐Arg+DOX+ICG supramolecular nanoparticles, the heating curves were recorded during six heating/cooling cycles (10 min laser irradiation followed with 10 min natural cooling) with 808 nm laser irradiation. It has been found that no significant attenuation in photothermal performance was observed for all the three nanomaterials, indicating their outstanding photothermal stability (Figure S18, Supporting Information). Moreover, on the basis of the heating and cooling curves, the photothermal conversion efficiency of WP5⊃D/DL/L‐Arg+DOX+ICG was calculated to be 62.60%, 62.01%, and 62.91% according to the previous literature (Figure S19, Supporting Information),^[^ ^37^, ^38^ ^]^ which further proved their excellent and supramolecular chirality‐independent photothermal performance.

With high drug encapsulation efficiency, the release behavior of DOX from the WP5⊃D/L‐Arg+DOX+ICG supramolecular nanoparticles was investigated. It has been found that under physiological condition (pH 7.4), the cumulative release of DOX within 24 h was solely 13.4% and 13.8% for WP5⊃D‐Arg+DOX+ICG and WP5⊃L‐Arg+DOX+ICG (Figure S20a,b, Supporting Information). While at pH 6.5, which mimics the tumor acidic microenvironment, the release efficiency reached ≈30% in both cases, indicating their potential for tumor‐site targeted drug release (Figure S20a,b, Supporting Information). Furthermore, to clarify the high drug release mechanism at acidic condition, the behavior of the WP5⊃D/L‐Arg+DOX+ICG nanoparticles at pH 6.5/5.5 was investigated. From Figure S21 (Supporting Information), the diameters of the WP5⊃D/DL/L‐Arg+DOX+ICG nanoparticles were evidently decreased at pH 6.5/5.5, confirming the destruction of their initial core‐shell nanostructures, which can be caused by the partially protonation of WP5. The nanostructure disruption at acidic condition contributed to the higher DOX release efficiency. In addition, upon 808 nm laser irradiation, even rapid release of DOX was observed and the corresponding release efficiency was calculated to be 42.4% and 42.5% for WP5⊃D/L‐Arg+DOX+ICG within 6 h (Figure S20c,d, Supporting Information), which is ≈3 times higher than that in the absence of irradiation. As a consequence, the pH and NIR light dual responsive drug release behavior makes WP5⊃Arg+DOX+ICG excellent candidates for chemotherapy.

### 2.4 Chirality‐Dependent Anti‐Tumor Effect In Vitro

**Figure 5:**
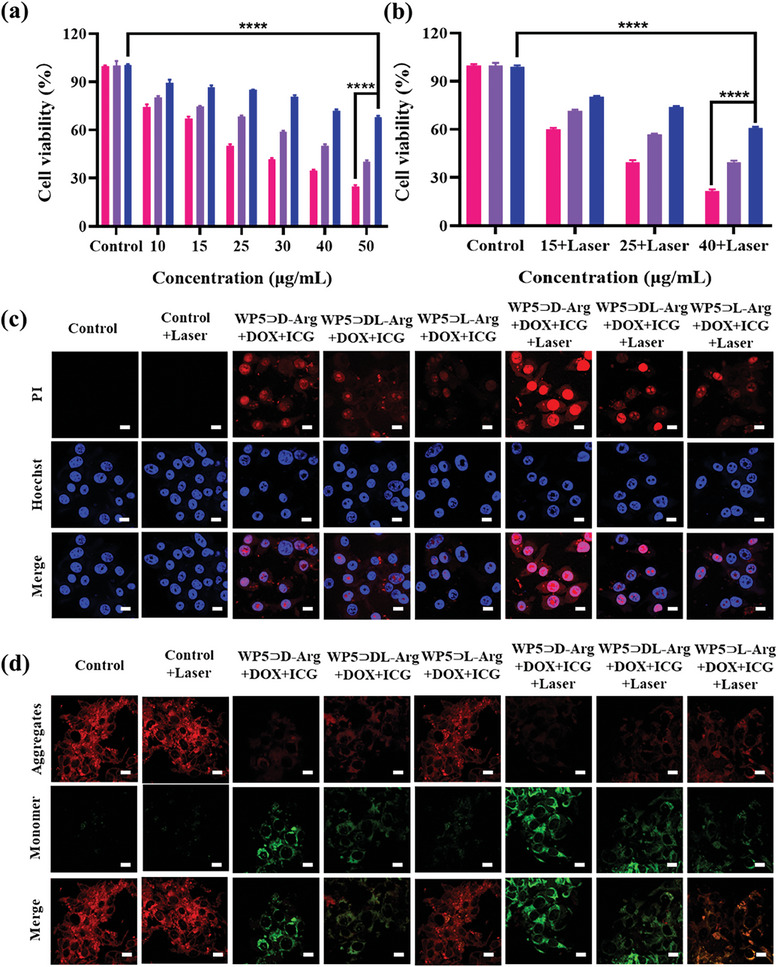
a) Relative viability of CT26 cell after being incubated with various concentrations of WP5⊃D/DL/L‐Arg+DOX+ICG. Red: WP5⊃D‐Arg+DOX+ICG, Purple: WP5⊃DL‐Arg+DOX+ICG, Blue: WP5⊃L‐Arg+DOX+ICG. b) Relative viability of CT26 cell after being incubated with various concentrations of WP5⊃D/DL/L‐Arg+DOX+ICG upon 808 nm laser irradiation (1.0 W cm^−2^). Red: WP5⊃D‐Arg+DOX+ICG, Purple: WP5⊃DL‐Arg+DOX+ICG, Blue: WP5⊃L‐Arg+DOX+ICG. c) Confocal fluorescence images of Hoechst and PI co‐stained CT26 cells with various treatments. For Hoechst channel: λ_Ex_ = 405 nm, λ_Em_ = 410–500 nm. For PI channel: λ_Ex_ = 543 nm, λ_Em_ = 605–700 nm. d) Confocal fluorescence images of JC‐1‐stained CT26 cells with various treatments. Scale bars: 10 µm. Data were presented as mean ± SD (*n* = 3). *p*‐value: **** *p* < 0.0001.

To evaluate the biocompatibility, the in vitro cytotoxicity of the as prepared supramolecular nanoparticles toward L02 (normal liver cells) was studied using CCK‐8 assay. As revealed from Figure S22 (Supporting Information), no evident cell death was observed for WP5⊃D/DL/L‐Arg and WP5⊃D/DL/L‐Arg+DOX+ICG, indicating their excellent biocompatibility in the concentration range of 10 to 100 µg mL^−1^. Subsequently, the anti‐tumor effect of WP5⊃D/DL/L‐Arg and WP5⊃D/DL/L‐Arg+DOX+ICG was investigated. As shown in Figure S23 (Supporting Information), the WP5⊃D‐Arg, WP5⊃DL‐Arg and WP5⊃L‐Arg exhibited no obvious cytotoxicity to CT26 cells. However, the WP5⊃D‐Arg+DOX+ICG, WP5⊃DL‐Arg+DOX+ICG, and WP5⊃L‐Arg+DOX+ICG were demonstrated with distinct cytotoxicity toward CT26 cells at the same condition by CCK‐8 results (**Figure** **5**a), which can be due to the DOX release induced chemotherapy. The above assumption was further confirmed by the lower cell viability of WP5⊃D/DL/L‐Arg+DOX (Figure S24a, Supporting Information). Upon carefully inspecting the aforementioned results, interestingly, we found that the cytotoxicity of WP5⊃D/DL/L‐Arg related supramolecular nanoparticles was chirality‐dependent. When incubated with CT26 cells, the supramolecular nanoparticles in the D‐form consistently induced the highest percentage of cell death, whereas the supramolecular nanoparticles in the L‐form consistently exhibited the lowest cytotoxicity toward CT26 cells at the same condition.

To further evaluate the chirality‐specific photothermal therapeutic activity of the supramolecular nanoparticles, the cell viability of CT26 cells treated with WP5⊃D/DL/L‐Arg+DOX+ICG upon 808 nm laser irradiation was investigated. As can be seen from Figure 5b, at various concentrations, the cell viability was significantly decreased in the presence of laser irradiation, indicating the outstanding chemo‐photothermal synergistic performance of WP5⊃D/DL/L‐Arg+DOX+ICG in vitro. Additionally, it is apparent that in these cases, the percentage of cell death follows a specific order of WP5⊃D‐Arg+DOX+ICG>WP5⊃DL‐Arg+DOX+ICG>WP5⊃L‐Arg+DOX+I CG. The same trends were observed when the WP5⊃D/DL/L‐Arg+ICG nanoparticles were employed as photothermal agents (Figure S24b, Supporting Information). To more intuitively determine the cell viability of WP5⊃D/DL/L‐Arg+DOX+ICG nanoparticles, the CT26 cells with various treatments were co‐stained with Hoechst (blue fluorescence) and PI (red fluorescence, for dead cells). As shown from Figure 5c, the groups with laser irradiation induced higher cell death in comparison with the ones in the absence of irradiation. In addition, in each case, the WP5⊃D‐Arg+DOX+ICG exhibited enhanced anti‐tumor effect than WP5⊃DL‐Arg+DOX+ICG, followed by WP5⊃L‐Arg+DOX+ICG. In particular, with 808 nm laser irradiation, the WP5⊃D‐Arg+DOX+ICG caused almost complete death of CT26 cells, indicating their superior chemo‐photothermal synergistic therapy. Taken together, all the above results clearly indicated that the anti‐tumor effect of WP5⊃D/DL/L‐Arg+DOX+ICG was chirality‐dependent, which can be ascribed to the differentiated cellular uptake efficiency of WP5⊃D/DL/L‐Arg+DOX+ICG toward cells as aforementioned.

Since the decrease in mitochondrial membrane potential (ΔΨm) is a hallmark event of cell apoptosis, the mitochondrial membrane potential was monitored using JC‐1 probe to understand the cell death mechanism caused by WP5⊃D/DL/L‐Arg+DOX+ICG. It is text knowledge that the fluorescence of JC‐1 can be varied from red (aggregated state) to green (monomer state) along with the decreasing of the mitochondrial membrane potential. From Figure 5d, it is clear that enhanced green fluorescence was observed for all the experimental groups in contrast with the control group, confirming the decreasing of the ΔΨm and the mitochondrial pathway of apoptosis. Moreover, either in the case without or with laser irradiation, the WP5⊃D‐Arg+DOX+ICG nanoparticles treated cells displayed the strongest green fluorescence, which further indicated the most significant anti‐tumor effect of D‐formed nanomaterials. Since the anti‐tumor effect of WP5⊃D‐Arg+DOX+ICG nanoparticles functions through the dysfunction of intracellular mitochondrial, the better anti‐tumor effect of D‐formed nanoparticles is undoubtedly caused by their higher cellular internalization efficiency.

### 2.5 Chirality‐Dependent Anti‐Tumor Effect In Vivo

**Figure 6:**
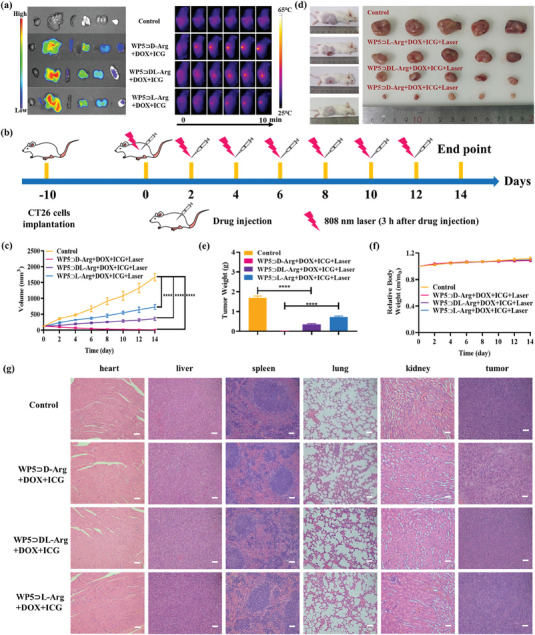
a) (Left) Ex vivo fluorescence images of major organs and tumor dissected from CT26 tumor bearing mice taken at 3 h i.v. injection of WP5⊃D/DL/L‐Arg+DOX+ICG. (Right) The IR thermal images of CT26 tumor‐bearing mice treated with WP5⊃D/DL/L‐Arg+DOX+ICG. b) Overall schedule of the in vivo anti‐tumor experiments. c) Tumor volume of WP5⊃D/DL/L‐Arg+DOX+ICG treated mice at different periods. Data were presented as mean ± SD (*n* = 5). d) Photographs of tumors collected from WP5⊃D/DL/L‐Arg+DOX+ICG treated mice on day 14. e) Tumor weight of WP5⊃D/DL/L‐Arg+DOX+ICG treated mice on day 14. Data were presented as mean ± SD (*n* = 5). f) Relative bodyweight of WP5⊃D/DL/L‐Arg+DOX+ICG treated mice recorded at different periods. Data were presented as mean ± SD (n = 5). g) H&E staining images of heart, liver, spleen, lung, kidney, and tumor after various treatments for 14 days. Scale bars: 50 µm. *p*‐value: **** *p* < 0.0001.

Encouraged by the positive results from the in vitro study, subsequent in vivo experiments were conducted. Primarily, the blood biochemical analysis was performed to evaluate the in vivo cytotoxicity of the WP5⊃D/DL/L‐Arg+DOX+ICG supramolecular nanoparticles. According to the blood routine data of mice treated with the WP5⊃D/DL/L‐Arg+DOX+ICG (Figure S25, Supporting Information), no obvious changing of the major components, i.e., RBC, HGB, HCT, MCV, and MCH, was observed. Subsequently, after 3 h injection of WP5⊃D/DL/L‐Arg+DOX+ICG, the major organs and tumors of CT26 tumor‐bearing mice with various treatments were dissected. From the corresponding ex vivo fluorescent images (**Figure** **6**a), the tumor accumulation of WP5⊃D‐Arg+DOX+ICG was significantly higher than that of WP5⊃DL‐Arg+DOX+ICG and WP5⊃L‐Arg+DOX+ICG, confirming the chirality‐specific performance in vivo. As the WP5⊃D/DL/L‐Arg+DOX+ICG nanoparticles were largely accumulated in liver, the content of ALP, ALT, and AST in serum was measured and no evident changing was obtained for the WP5⊃D/DL/L‐Arg+DOX+ICG treated groups (Figure S26, Supporting Information), which demonstrated the negligible adverse effect on liver function.

To further assess the in vivo photothermal performance, the WP5⊃D/DL/L‐Arg+DOX+ICG nanoparticles were injected into the tail vein of the tumor‐bearing mice separately. Subsequently, the mice were anesthetized and exposed to 808 nm laser for 10 min. According to the infrared thermal images (Figure 6a), the temperature of the tumor site was increased along with the irradiation time for various treatments. However, the temperature of the WP5⊃D‐Arg+DOX+ICG treated tumor site reached 60 °C, whereas WP5⊃DL‐Arg+DOX+ICG and WP5⊃L‐Arg+DOX+ICG treated tumor sites were limited to 52 and 46 °C, respectively. Considering that the photothermal conversion efficiency of all the three types of nanoparticles were almost identical, the better photothermal performance of D‐formed nanoparticles in vivo is undoubtedly related to their chirality‐actuated higher tumor accumulation efficiency.

The in vivo anti‐tumor effect of WP5⊃D/DL/L‐Arg+DOX+ICG was eventually evaluated under 808 nm laser irradiation. The overall schedule of the in vivo anti‐tumor experiments is shown in Figure 6b. Specifically, the tumor‐bearing mice were divided into 4 groups and treated with PBS, WP5⊃D‐Arg+DOX+ICG, WP5⊃DL‐Arg+DOX+ICG, and WP5⊃L‐Arg+DOX+ICG, respectively. For the WP5⊃D/DL/L‐Arg+DOX+ICG treated groups, the tumor sites of mice were exposed to 808 nm laser (1.0 W cm^−2^) for 10 min at 3 h injection. During the treatment period, the tumor volumes were recorded every two days. As shown in Figure 6c and Figure S27 (Supporting Information), the tumor grew rapidly for the control group and the tumor growth inhibition effect was observed for all the three experimental groups, providing further evidence for the antitumor activity of the WP5⊃D/DL/L‐Arg+DOX+ICG through chemo‐photothermal synergistic therapeutic manner. What is more, in contrast with the tumor volume of the control group, the tumor inhibition rate of WP5⊃D‐Arg+DOX+ICG was calculated to be 99.4% after 14 days’ treatment, which is much higher than that of WP5⊃DL‐Arg+DOX+ICG (78.6%) and WP5⊃L‐Arg+DOX+ICG (56.4%). The tumor photographs and weight after 14 days’ therapy further confirmed the strongest anti‐tumor activity of WP5⊃D‐Arg+DOX+ICG (Figure 6d,e) due to their highest accumulation efficiency in tumor site. Despite the efficient tumor inhibition efficiency was achieved, the growing of the body weight (Figure 6f) and the negligible damage of the key organs (i.e., heart, liver, spleen, lung, and kidney) according to H&E stained images (Figure 6g) were observed, confirming the ignorable adverse effect. The above results well proved that supramolecular chirality is a crucial factor in biotherapy and can be manipulated to maximize cancer therapeutic efficacy.

## 3 Conclusion

In this study, we explored the effect of supramolecular chirality on cancer therapy. Supramolecular chiral chemo‐photothermal agents (WP5⊃D/L‐Arg+DOX+ICG) with the chirality transfer from D/L‐Arg to WP5 have been constructed through host‐guest interaction. The almost identical drug encapsulation efficiency, drug release behavior and photothermal performance of WP5⊃D/L‐Arg+DOX+ICG in aqueous solution were demonstrated. However, due to the chirality‐specific cellular uptake efficiency of WP5⊃D/L‐Arg+DOX+ICG, the differential tumor accumulation efficiency and photothermal conversion in biological system were observed. In all the above cases, WP5⊃D‐Arg+DOX+ICG showed evidently better performance than WP5⊃L‐Arg+DOX+ICG. Eventually, the significantly superior anti‐tumor effect of WP5⊃D‐Arg+DOX+ICG (inhibition rate: 99.4%) over WP5⊃L‐Arg+DOX+ICG (inhibition rate: 56.4%) was confirmed as result of supramolecular chirality‐amplified cellular uptake efficiency. Our findings provide valuable insight into the role of supramolecular chirality in cancer therapy and offer direct guidance for the development of cancer therapeutic agents.

## Conflict of Interest

The authors declare no conflict of interest.

## Supporting information

Supporting Information

## Data Availability

The data that support the findings of this study are available from the corresponding author upon reasonable request.
