# Supplementary material for: Supramolecular Chiral Binding Affinity‐Achieved Efficient Synergistic Cancer Therapy
Source: Adv Sci (Weinh). 2024 Feb 21;11(16):2308493. doi: 10.1002/advs.202308493 (PMC11040370; doi:10.1002/advs.202308493)
Supplement: Supplementary file 1 — Supporting Information [file ADVS-11-2308493-s001.pdf]

ADVANCED SCIENCE
Open Access

Supporting Information

for *Adv. Sci.*, DOI 10.1002/advs.202308493

Supramolecular Chiral Binding Affinity-Achieved Efficient Synergistic Cancer Therapy

Jinfeng Zhou, Jiake Gu, Xiaohuan Sun, Qianyun Ye, Xuan Wu*, Juqun Xi, Jie Han* and Yu Liu**

Supporting Information

for

Supramolecular chiral binding affinity-achieved efficient synergistic cancer therapy

Jinfeng Zhou,[a] Jiake Gu,[b] Xiaohuan Sun,*[a] Qianyun Ye,[a] Xuan Wu,*[a] Juqun Xi,[b] Jie Han,*[a] Yu Liu*[c]

[a] School of Chemistry and Chemical Engineering, Yangzhou University, Yangzhou, Jiangsu 225002, China

[b] School of Medicine, Yangzhou University, Yangzhou, Jiangsu 225002, China.

[c] College of Chemistry, State Key Laboratory of Elemento-Organic Chemistry, Nankai University, Tianjin 300071, China

Methods

Materials. The D/DL/L-Arg, DOX, ICG and soy lecithin were purchased from Energy Chemical Reagent Co. Ltd. The other reagents were provided by Sinopharm Chemical Reagent Co. Ltd. The deionized water was filtered through the Milli-Q Plus system (Millipore, France). CT26 cells were purchased from the experimental cell resource center of the Shanghai Institutes for Biological Sciences.

Instruments. The morphology of the as-prepared nanomaterials was investigated by the TEM technique (HT7800 Hitachi Co., Japan). The chirality of the as-prepared nanomaterials was measured by Circular Dichroism spectrapolarimeter J-810 (JASCO Co, Janpan). The UV-Vis spectra were measured in the range between 200 and 800 nm on UV2550 spectrometer (SHIMADZU Co., Japan). ^{1}H NMR spectra were collected at 298 K on Agilent DD2 400 MHz spectrometer. DLS was performed with ZEN3690 zetasizer. The relative cell viability was calculated according to the UV-Vis measurement by a microplate reader (Tecan Spark). The photothermal experiments were performed with 808 nm-laser (Changchun Laser Technology Co., Ltd.). The heat caused by the interaction between WP5⊃Arg and lipid bilayers were measured by Nano ITC (TA, America).

Synthesis of WP5. WP5 was synthesized according to the previous publication.[S1]

Preparation of WP5⊃D/DL/L-Arg solution. To prepare WP5⊃D/DL/L-Arg solution, 1.0 mg (0.0057 mmol) of D/DL/L-Arg was dissolved in 2.0 mL of ultrapure water, respectively, followed with the addition of 8.1 mg (0.0057 mmol) of WP5 to the D/DL/L-Arg solution. Subsequently, the above mixtures were ultrasonicated. After

that, the pH of each solution was adjusted to 7.4. The as prepared WP5⊃D/DL/L-Arg solutions were stored at room temperature for future use.

Preparation of liposome. Liposomes were prepared by thin film dispersion. Specifically, soy lecithin and cholesterol (5:1 mass ratio) were dissolved in chloroform. The solvent is then removed with a rotary evaporator. Subsequently, 10 mL of ultrapure water was added, and the solution was stirred at 52 °C for 3 hours. After that, the solution was ultrasonicated for 10 min, resulting the liposome dispersion.

Preparation of WP5⊃D/DL/L-Arg+DOX. To prepare WP5⊃D/DL/L-Arg+DOX, DOX (molar ratio of DOX:WP5⊃D/DL/L-Arg = 1:2) was added to 10 mL of WP5⊃D/DL/L-Arg solution, respectively, and stirred for 24 hours. After that, WP5⊃D/DL/L-Arg+DOX solution was loaded into dialysis bag (molecular weight cutoff 14000), and was dialyzed in ultrapure water to remove the unloaded DOX. The concentration of WP5⊃D/DL/L-Arg+DOX mentioned in this work is referred to the concentration of Arg.

Preparation of WP5⊃D/DL/L-Arg+ICG. To prepare WP5⊃D/DL/L-Arg+ICG, ICG (molar ratio of ICG:WP5⊃D/DL/L-Arg = 1:2) was added to 10 mL of WP5⊃D/DL/L-Arg solution, respectively, and stirred for 24 hours. After that, WP5⊃D/DL/L-Arg+ICG solution was loaded into dialysis bag (molecular weight cutoff 14000), and was dialyzed in ultrapure water to remove the unloaded ICG. The concentration of WP5⊃D/DL/L-Arg+ICG mentioned in this work is referred to the concentration of Arg.

Preparation of WP5⊃D/DL/L-Arg+DOX+ICG. To prepare WP5⊃D/DL/L-

Arg+DOX+ICG, DOX and ICG (molar ratio of DOX: ICG: WP5⊃D/DL/L-Arg = 1:1:2) were added to 10 mL of WP5⊃D/DL/L-Arg solution, respectively, and stirred for 24 hours. After that, WP5⊃D/DL/L-Arg+DOX+ICG solution was loaded into dialysis bag (molecular weight cutoff 14000), and was dialyzed in ultrapure water to remove the unloaded DOX and ICG. The aqueous solution outside the dialysis bag was changed periodically until the aqueous solution outside the dialysis bag showed negligible UV-Vis absorption of both DOX and ICG. The aqueous solution outside the dialysis bag was collected and measured by UV-Vis spectrometer. Then, the encapsulated amounts of DOX and ICG were calculated according to the standard calibration curve (y_{DOX} = 0.01771x + 0.01448, y_{ICG} = 0.11401x + 0.11733). The drug encapsulation efficiency of DOX and ICG loaded in WP5⊃D/DL/L-Arg were calculated according to the following formula: Encapsulation efficiency = mass of loaded drug/total amount of drug input. The concentration of WP5⊃D/DL/L-Arg+ICG mentioned in this work is referred to the concentration of Arg.

DOX releasing behavior. To study the release behavior of DOX, 3 mL of WP5⊃D/L-Arg+DOX+ICG solution was placed into a dialysis bag, and then the dialysis bag was placed into a 50 mL centrifuge tube, which was pre-filled with 30 mL PBS buffer solution (pH 7.4 or 6.5). The centrifuge tube was kept shaking by a constant temperature shaker at 37 °C and 118 rpm. At various time periods, the UV-Vis absorption of DOX at 478 nm and ICG at 780 nm of the solution outside the dialysis bag were determined by UV spectrophotometer. After the measurement, the solution was poured back into the centrifuge tube. To study the drug release behavior under NIR light irradiation, the

above experiments were repeated under 808 nm laser irradiation. The releasing efficiency of DOX was calculated according to the standard calibration curve of DOX in PBS. (pH = 7.4: $y_{DOX} = 0.01642x - 0.00247$; pH = 6.5: $y_{DOX} = 0.01640x - 0.02188$,)

Cellular uptake behavior. To investigate the effect of supramolecular chirality of WP5⊃D/DL/L-Arg+DOX+ICG on cellular uptake, CT26 cells were inoculated onto a confocal culture dish for 12 hours. Subsequently, WP5⊃D/DL/L-Arg+DOX+ICG were added. After 24 hours, the cells were washed with PBS for three times and the nuclei were stained with DAPI. Eventually, confocal fluorescence microscopy was used to observe the uptake behavior of WP5⊃D/DL/L-Arg+DOX+ICG.

To explore the cellular uptake mechanism of WP5⊃D/DL/L-Arg+DOX+ICG into CT26 cells, CT26 cells were seeded on a confocal dish for 12 hours, and then placed in a refrigerator at 4 °C for 1 hour, Then WP5⊃D/DL/L-Arg+DOX+ICG were added and continued to incubate in a refrigerator at 4 °C for 24 hours. The control group was also added with the same concentration of WP5⊃D/DL/L-Arg+DOX+ICG, and incubated at 37 °C for 24 hours. After incubation, the cells were washed three times with PBS and the nuclei were stained with DAPI for 20 minutes. The cellular uptake results were then observed by confocal fluorescence microscopy.

To quantify the mean fluorescence intensity of WP5⊃D/DL/L-Arg+DOX+ICG treated CT26 cells by flow cytometry, the CT26 cells were inoculated in a 6-well plate at a density of 1×10^5 cells per well at 37 °C and 5% CO_2 for 24 hours. Then, the CT26 cells were incubated with WP5⊃D/DL/L-Arg+DOX+ICG (40 μg mL^{-1}, 37 °C, 24 hours), and the cell culture medium was sucked up 24 hours later followed with PBS

washing. The cells with various treatments were then examined by flow cytometry. Total 20000 cells were counted via FITC channel. The mean fluorescence intensity was analyzed by FlowJo.

Chlorpromazine (an inhibitor of clathrin-mediated endocytosis), nystatin (an inhibitor for caveolin-dependent endocytosis), and wortmannin (a macropinocytosis inhibitor) were selected to investigate the endocytosis mechanisms. Firstly, CT26 cells were seeded on a confocal dish for 12 hours, and then incubated with corresponding inhibitors (chlorpromazine 20 μg mL^{-1}, nystatin 20 μg mL^{-1}, Wolman penicillin 1 μg mL^{-1}) for 1 h. Then WP5⊃D/DL/L-Arg+DOX+ICG was added and the mixture was incubated at 37 °C for 24 hours. After incubation, the cells were washed three times with PBS and the nuclei were stained with DAPI for 20 minutes. The cellular uptake results were then observed by confocal fluorescence microscopy.

Photothermal performance. WP5⊃D/DL/L-Arg+DOX+ICG solutions were exposed to 808 nm laser for 10 minutes, and the temperature changes of WP5⊃D/DL/L-Arg+DOX+ICG solutions were monitored separately by a thermocouple thermometer. To investigate the photothermal stability of WP5⊃D/DL/L-Arg+DOX+ICG, WP5⊃D/DL/L-Arg+DOX+ICG solution (500 μg mL^{-1}) were irradiated with an 808 nm laser for 10 minutes, then the laser was turned off and the solution was cooled at room temperature for 10 minutes. Six laser on/off cycles were performed.

To visually detect the photothermal behavior, upon the 808 nm laser irradiation, the temperature of WP5⊃D/DL/L-Arg+DOX+ICG solution was monitored by a thermal imager (Fluke, Ti-959 Hz). The thermal images were taken every 2 minutes.

Cytotoxicity tests. The cytotoxicity of WP5⊃D/DL/L-Arg, WP5⊃D/DL/L-Arg+DOX, WP5⊃D/DL/L-ICG and WP5⊃D/DL/L-Arg+DOX+ICG were evaluated by the CCK-8 method. Specifically, the CT26 cells were inoculated in a 96-well plate at a density of 1×10^4 cells per well at 37 °C and 5% CO_2 for 24 hours. Then, the cells were incubated with different concentrations of WP5⊃D/DL/L-Arg, WP5⊃D/DL/L-Arg+DOX, WP5⊃D/DL/L-ICG and WP5⊃D/DL/L-Arg+DOX+ICG (37 °C, 24 hours), respectively, for 24 hours. In the end, CCK-8 solution was added to each well, and the plates were incubated for another 4 hours. After that, a microplate reader was used to measure the UV-Vis absorbance at 450 nm.

Hoechst/PI Co-staining experiments. The antitumor activity of WP5⊃D/DL/L-Arg+DOX+ICG was evaluated by Hoechst/PI co-staining strategy. Specifically, the cell suspension was diluted and added to a 24 well plate where the cell slides have been placed. The cell density was 20000 cells per well. The above 24 well plate was incubated at 37 °C for 24 hours. Then the WP5⊃D/DL/L-Arg+DOX+ICG (50 μg mL^{-1}) were added and further incubated at 37 °C for 24 hours. For laser groups, after WP5⊃D/DL/L-Arg+DOX+ICG were added, The mixtures were exposed to 808 nm laser for 10 min and further incubated at 37 °C for 24 hours. After that, the cells of laser groups and other groups were stained with Hoechst/PI for 20 minutes, followed with the washing with sterile PBS for three times. Eventually, the cells on the slides were observed by confocal fluorescence microscopy.

Evaluation of cellular mitochondria potential. To monitor the mitochondrial membrane potential of CT26 cells after various treatments, the CT26 cells were

inoculated onto a confocal dish for 2 hours. After 24 hours of incubation with WP5⊃D/DL/L Arg+DOX+ICG, CT26 cells were stained with JC-1 at 37 °C for 20 minutes. After carefully washing according to the manufacturer's instructions, the CT26 cells on the slide were observed by confocal fluorescence microscopy.

***In vivo* chemo-photothermal cancer therapy**. Male Balb/c mice were purchased from Yangzhou University Comparative Medicine Centre. The *in vivo* experiments were authorized by the Science and Technology Department of Jiangsu Province and conducted with the approval of the Medical Ethics Committee of Yangzhou University Medical Academy (YXYLL-2022-44). To evaluate the cancer therapeutic efficacy of WP5⊃D/DL/L-Arg+DOX+ICG, four-week old male Balb/c mice were selected and fed until their body weight was greater than 20 g. Then, 100 μL of the cultured CT26 cell solution was piped and injected subcutaneously into the back of the mice. After one week, when the tumor volume increased to ~250 mm^3, the CT26 tumor bearing mice were divided into four groups (n = 5 in each group) and treated with the PBS, WP5⊃L-Arg+DOX+ICG+Laser, WP5⊃DL-Arg+DOX+ICG+Laser and WP5⊃D-Arg+DOX+ICG+Laser, respectively. In each case, 100 μL (500 μg/mL) of corresponding solution was injected into the tail vein of mice every two days. For groups 2, 3, and 4, after 3 hours' injection, the mice were subjected to 10 minutes' laser irradiation (808 nm, 1.0 W/cm^2) on the tumor area, respectively. Under NIR light irradiation, the temperature changes of tumor sites in each group of mice were monitored using an infrared thermal imager (Fluke, Ti-959 Hz). To measure the length and width of the tumor, a digital caliper was used. According to the equation: V (mm^3)

= length × width^2/2, the size of the tumor can be calculated. The tumor inhibition rate= $(V_{control} - V_{D/DL/L})/V_{control} \times 100\%$, $V_{control}$ refers to the average tumor volume in the control group on day 14, $V_{D/DL/L}$ refers to the average tumor volume in the WP5⊃D/DL/L-Arg+DOX+ICG group on day 14.

Histology Analysis. After 14 days of various treatments, the main organs (heart, liver, spleen, lungs, and kidneys) and tumors of the mice were removed and placed in PBS containing 4% paraformaldehyde. After that, the samples were embedded in paraffin, cut into 3 μm thickness, stained with hematoxylin and eosin (H&E), and then histopathology analysis was performed with optical microscope.

Statistical Analysis. The experimental results were expressed as mean ± SD. The significance of differences was analyzed using two-tailed unpaired student's t-test and one-way analysis of variance (ANOVA) test. Differences between datasets were considered to be significant at p value <0.05.

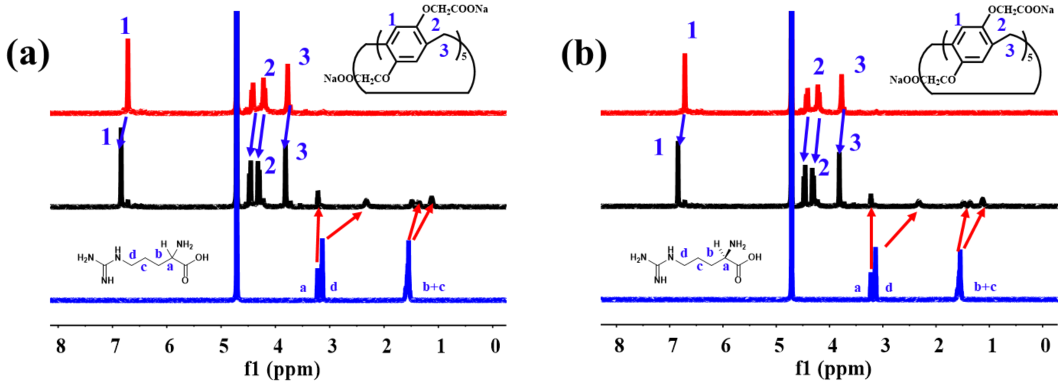

Fig. S1. (a) ^{1}H NMR spectra (400 MHz, D_2O, 298 K, pD = 7.2) of WP5, WP5⊃DL-Arg (molar ratio 1:1), and DL-Arg. (b) ^{1}H NMR spectra (400 MHz, D_2O, 298 K, pD = 7.2) of WP5, WP5⊃L-Arg (molar ratio 1:1), and L-Arg.

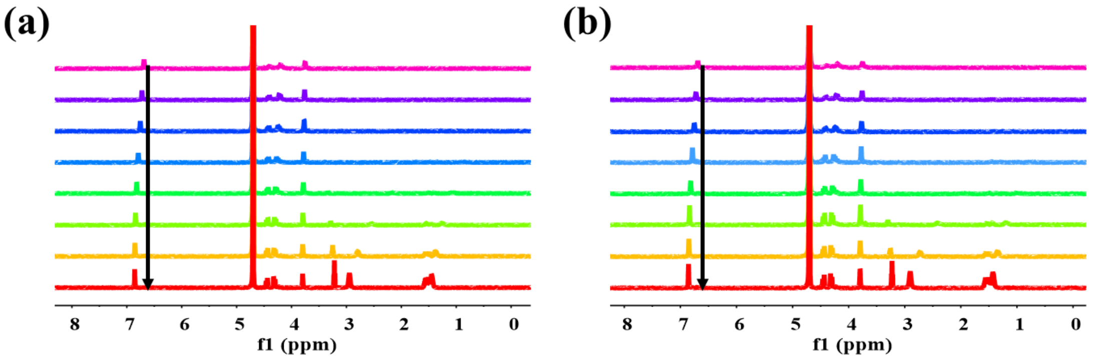

Fig. S2. ^{1}H NMR spectra (400 MHz, D2O, 298 K, pD = 7.2) of WP5 at a concentration of 1.0 mM upon addition of (a) DL-Arg and (b) L-Arg. From top to bottom, the concentration of DL-Arg or L-Arg was kept at 0, 0.1, 0.25, 0.5, 0.75, 1.25, 3.0, 5.0 mM.

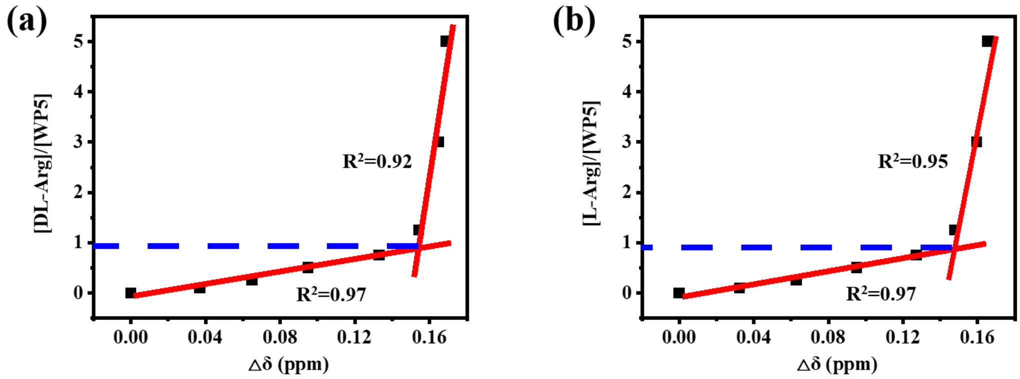

Fig. S3. Plot of the molar ratio of (a) [DL-Arg]/[WP5] and (b) [L-Arg]/[WP5] against the Δδ of H_1 of WP5.

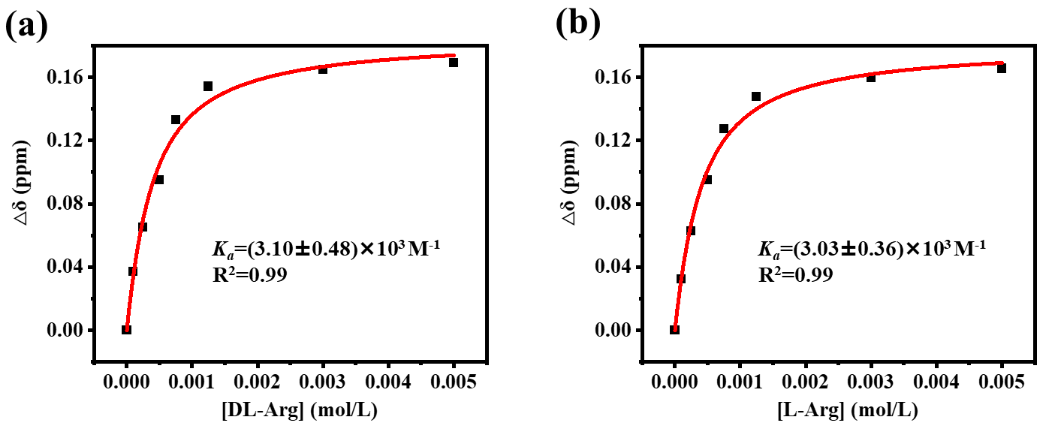

Fig. S4. Δδ of H_1 of WP5 against the concentration of (a) DL-Arg and (b) L-Arg. The red lines were obtained from non-linear curve-fitting.

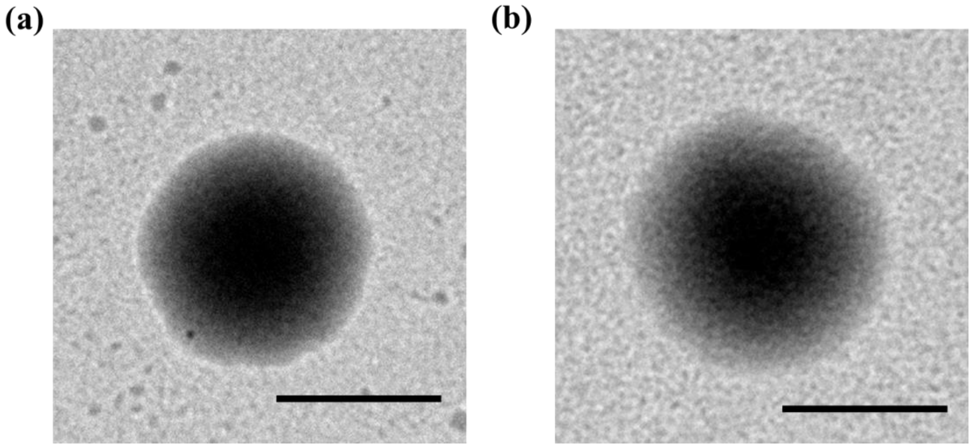

Fig. S5. The TEM image of (a) WP5⊃D-Arg and (b) WP5⊃L-Arg supramolecular nanoparticle showing the core-shell characteristic. Scale bars: 200 nm.

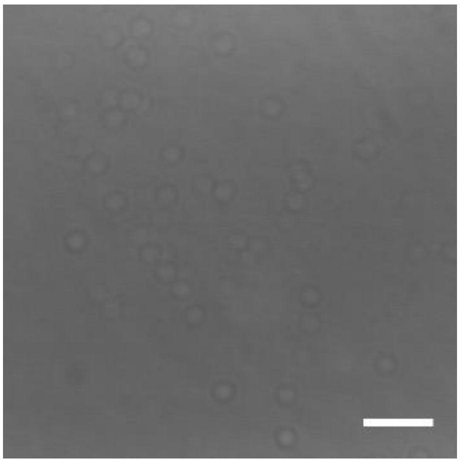

Fig. S6. Confocal fluorescent images of as synthesized liposomes. Scale bars: 5 μm.

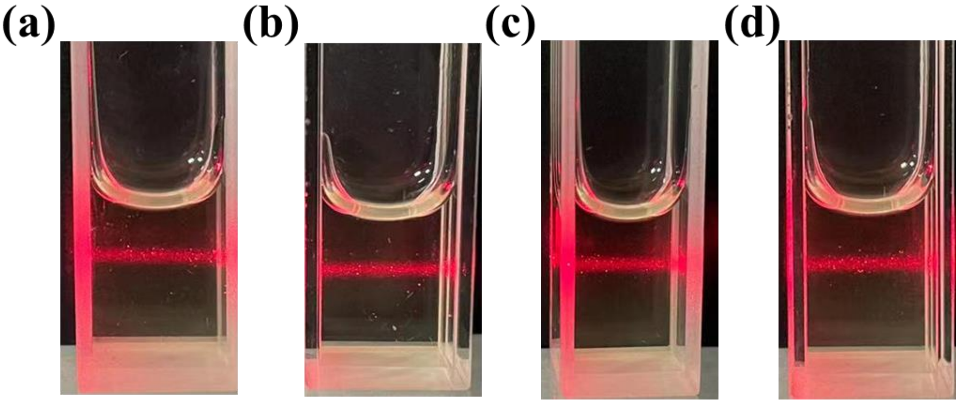

Fig. S7. The photographs showing the Tyndall effect of (a) WP5⊃D-Lys, (b) WP5⊃L-Lys, (c) WP5⊃D-Ala-Arg and (d) WP5⊃L-Ala-Arg.

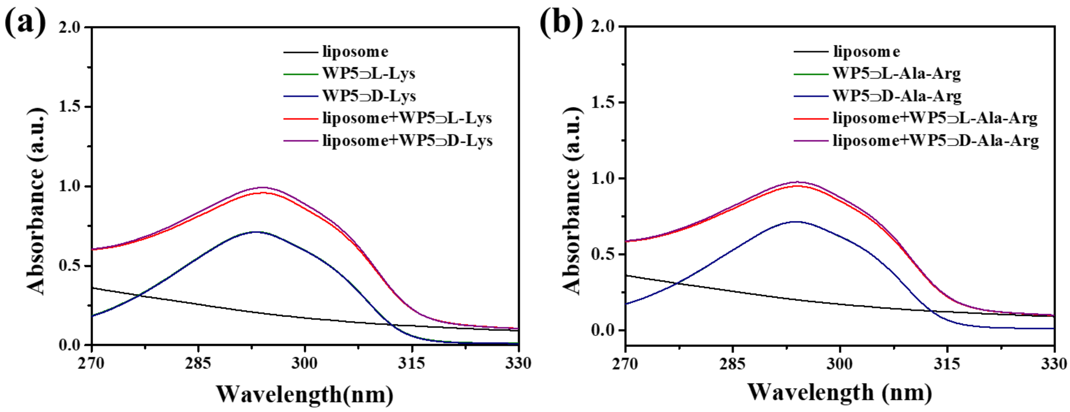

Figure S8. The UV-Vis spectra showing the interaction between (a) liposome and WP5⊃D/L-Lys and (b) liposome and WP5⊃D/L-Ala-Arg.

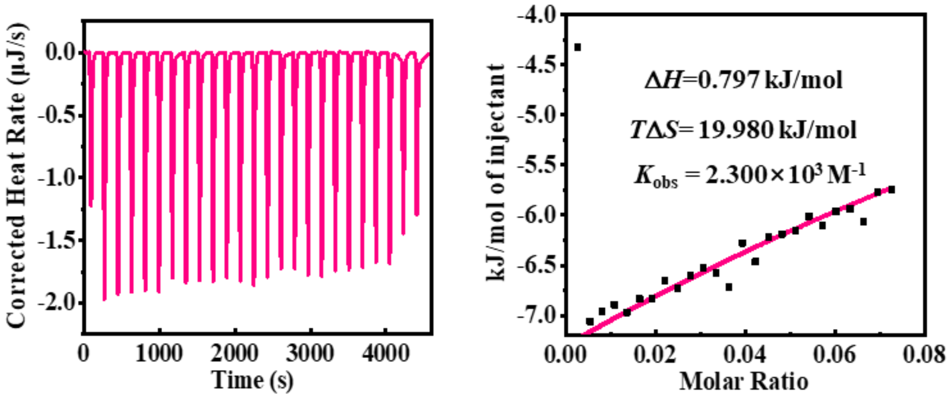

Fig. S9. ITC results of the addition of D-Arg into liposome dispersion.

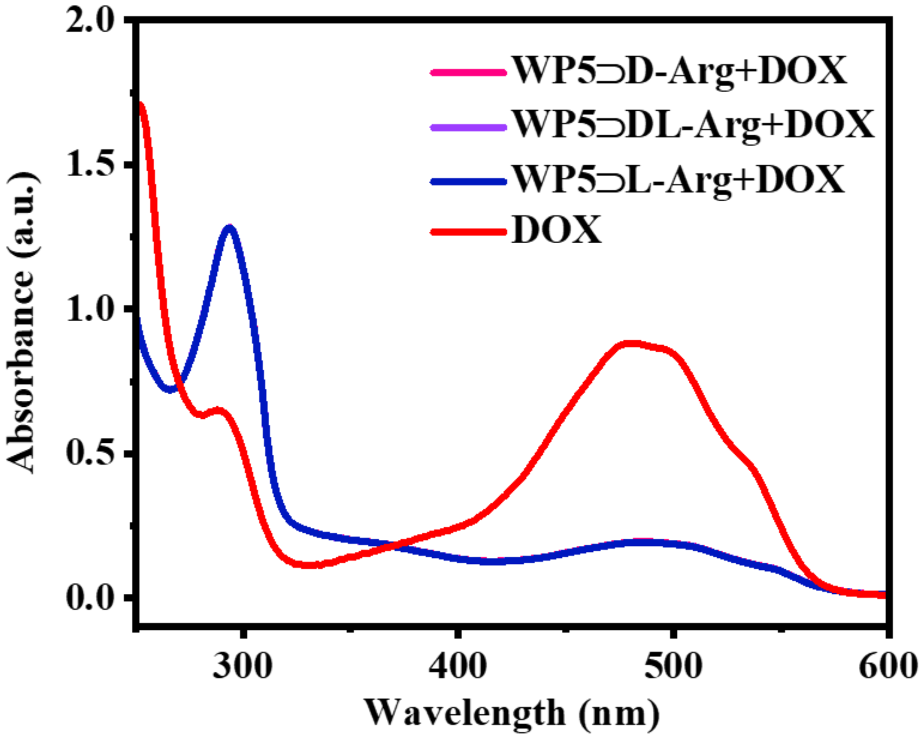

Fig. S10. UV-Vis spectra of WP5⊃D/DL/L-Arg+DOX and DOX aqueous solution.

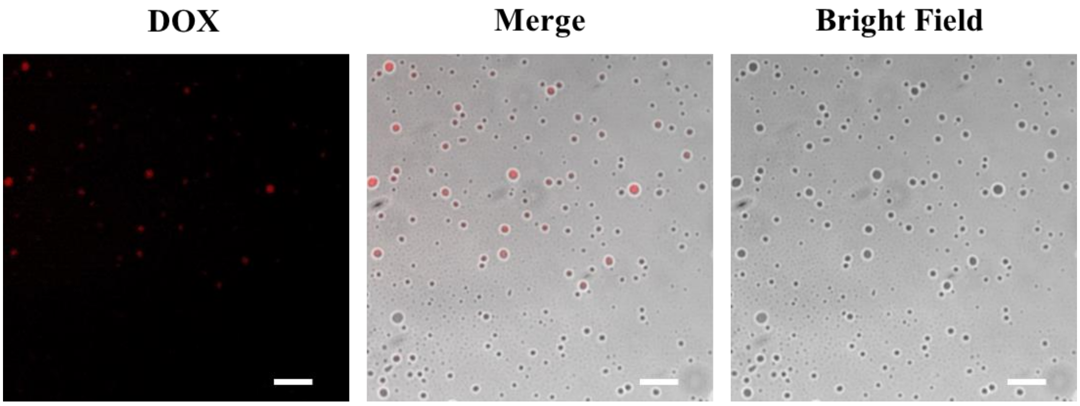

Fig. S11. Confocal fluorescence images of WP5⊃D-Arg+DOX. Scale bars: 5 μm.

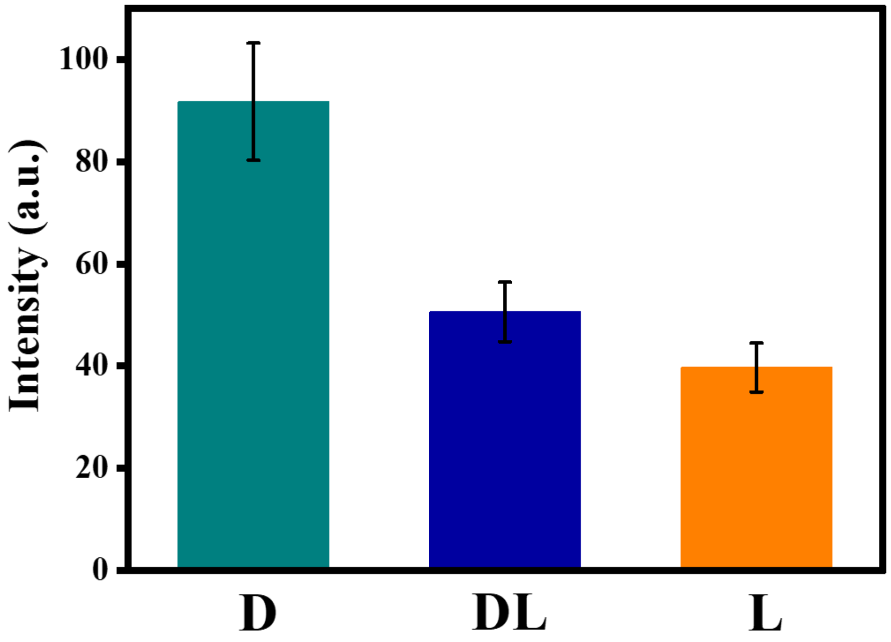

Fig. S12. The fluorescence intensity of the bilayers of liposomes treated with WP5⊃D/DL/L-Arg+DOX for 24 hours.

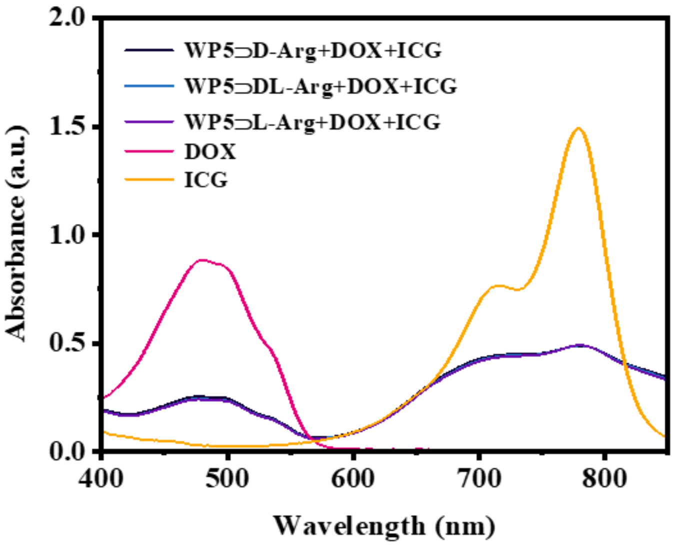

Fig. S13. UV-Vis spectra of WP5⊃D/DL/L-Arg+DOX+ICG, DOX and ICG.

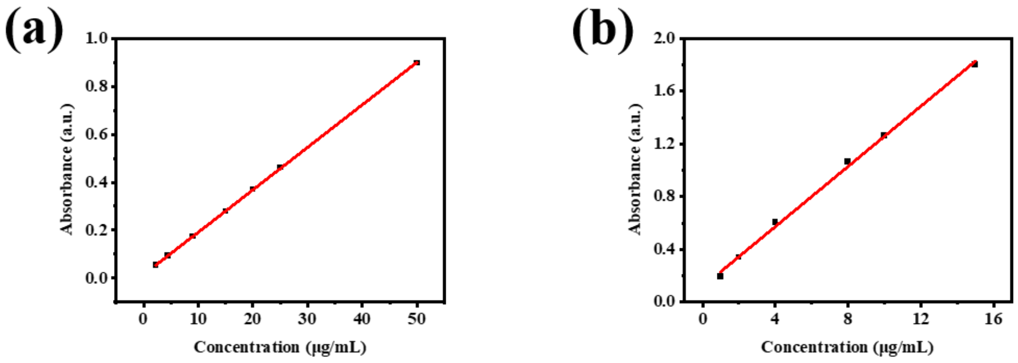

Fig. S14. The calibration curve of (a) DOX and (b) ICG aqueous solution.

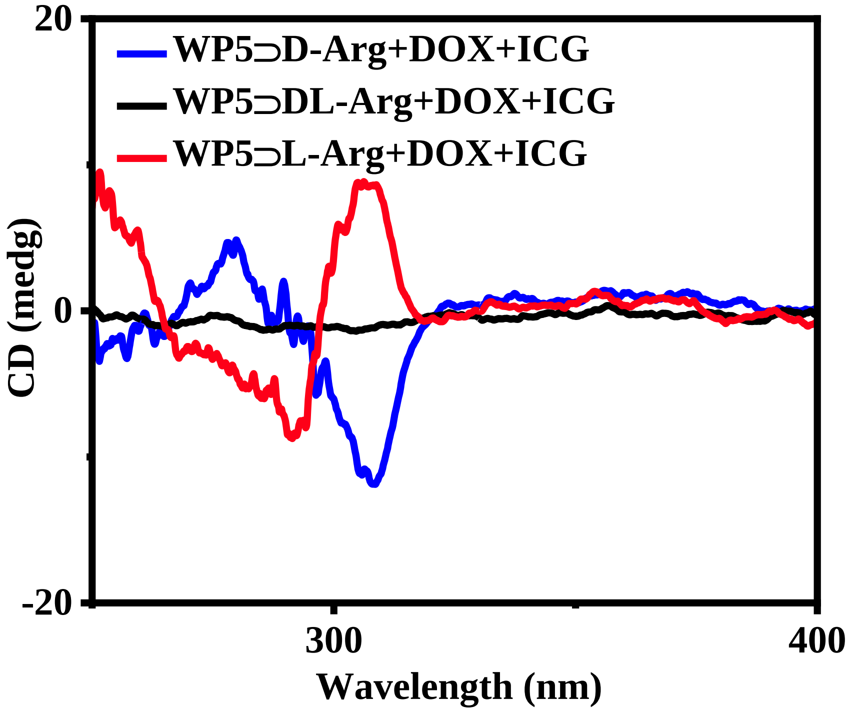

Fig. S15. The CD spectra of WP5⊃D/DL/L-Arg+DOX+ICG.

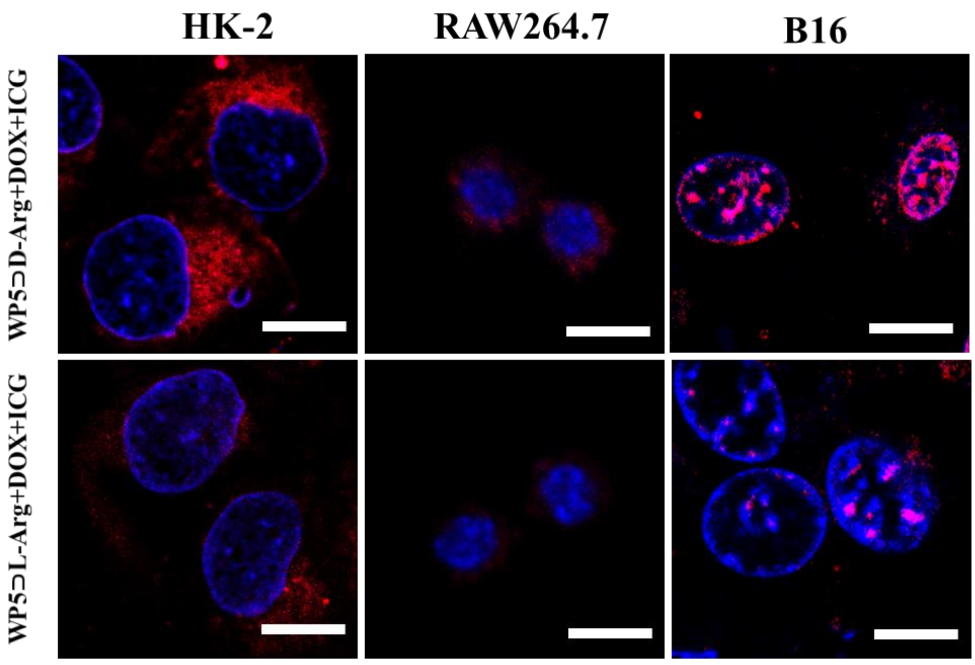

Fig. S16. Cellular internalization of WP5⊃D/DL/L-Arg+DOX+ICG (red) into DAPI-stained cell nuclei (blue) at 37 °C. Scale bars: 10 µm.

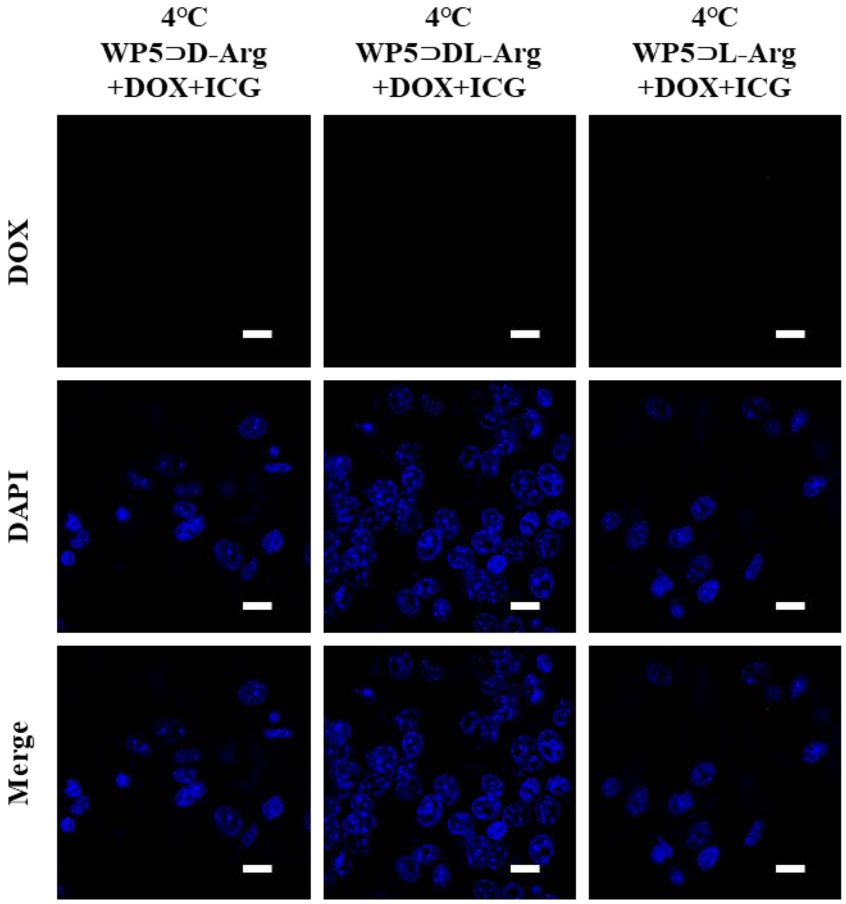

Fig. S17. Confocal images of DAPI-stained CT26 cells treated with WP5⊃D/DL/L-Arg+DOX+ICG at 4 °C. Scale bars: 10 μm.

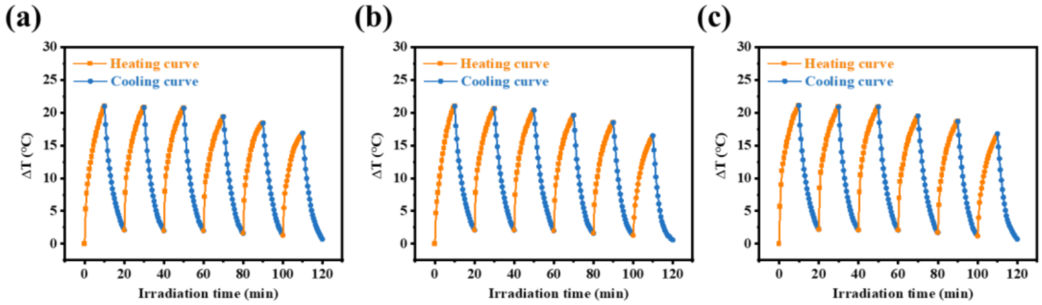

Fig. S18. Photostability test of (a) WP5⊃D-Arg+DOX+ICG, (b) WP5⊃DL-Arg+DOX+ICG and (c) WP5⊃L-Arg+DOX+ICG under the 808 nm laser irradiation (1.0 W/cm^2).

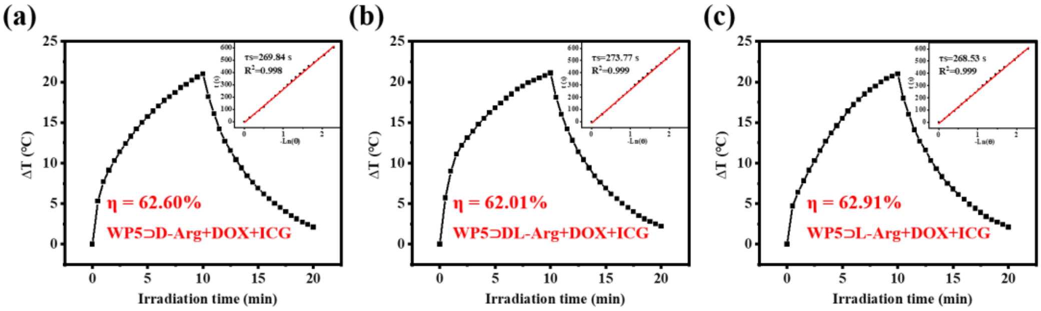

Fig. S19. (a-c) Heating and cooling curves of WP5⊃D/DL/L-Arg+DOX+ICG irradiated by 808 nm laser (1.0 W cm^{-2}) for 10 min, followed by natural cooling. The insets show the linear fitting of time from the cooling period versus the negative natural logarithm of driving force temperature for (a) WP5⊃D-Arg+DOX+ICG, (b) WP5⊃DL-Arg+DOX+ICG and (c) WP5⊃L-Arg+DOX+ICG.

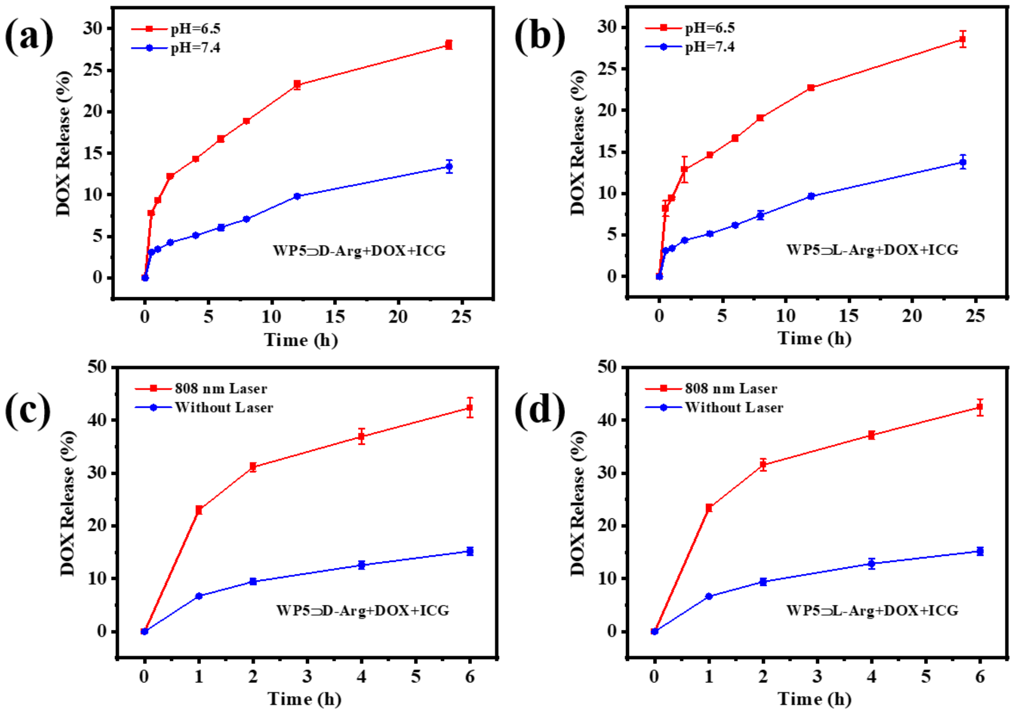

Fig. S20. The DOX releasing curve of (a) WP5⊃D-Arg+DOX+ICG and (b) WP5⊃L-Arg+DOX+ICG at different pH. The DOX releasing curve of (c) WP5⊃D-Arg+DOX+ICG and (d) WP5⊃L-Arg+DOX+ICG at pH 6.5 with or without 808 nm laser irradiation. Data were presented as mean ± SD (n = 3).

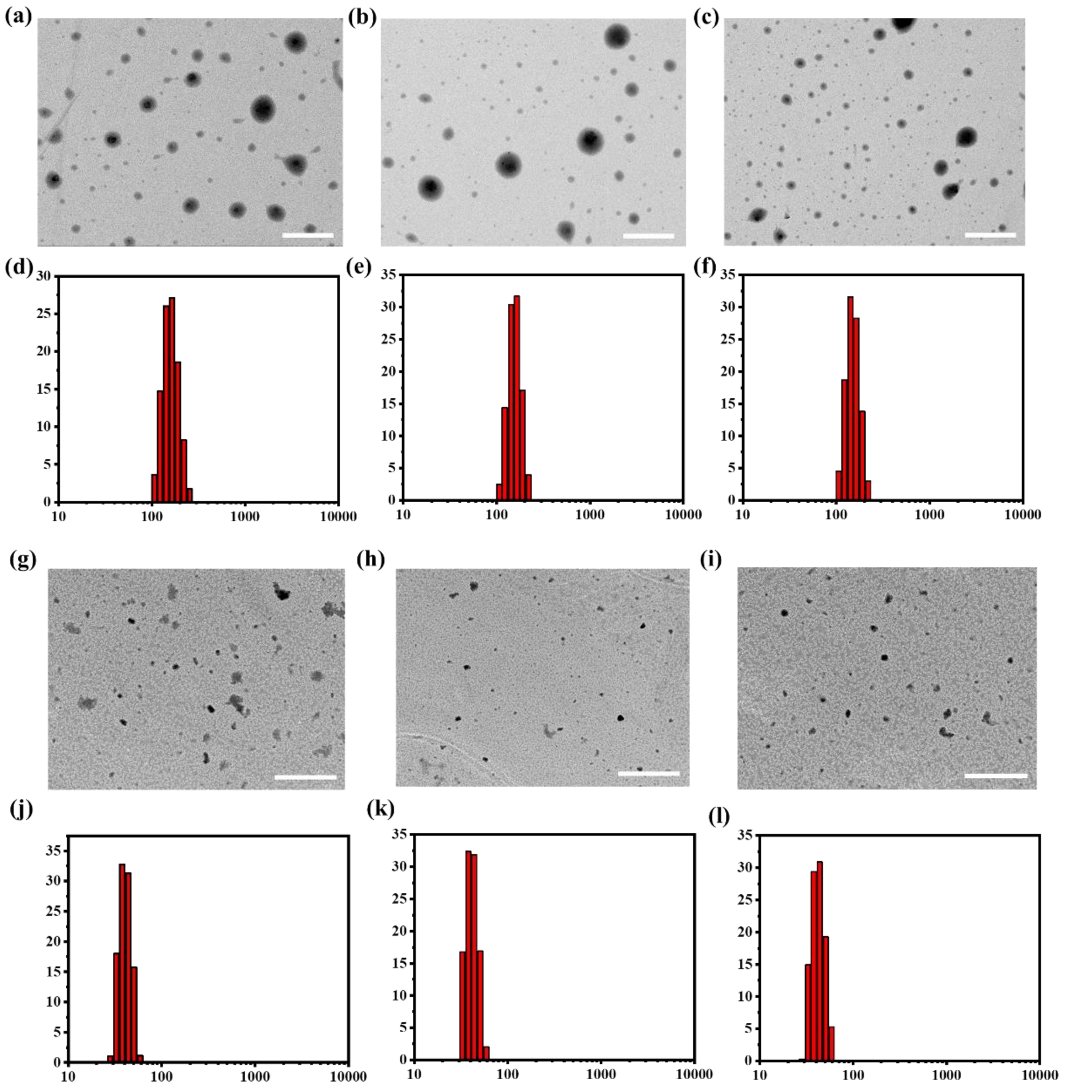

Fig. S21. The TEM and DLS characterization of (a, d) WP5⊃D-Arg+DOX+ICG, (b, e) WP5⊃DL-Arg+DOX+ICG and (c, f) WP5⊃L-Arg+DOX+ICG nanoparticles at pH 6.5. The TEM and DLS characterization of (g, j) WP5⊃D-Arg+DOX+ICG, (h, k) WP5⊃DL-Arg+DOX+ICG and (i, l) WP5⊃L-Arg+DOX+ICG nanoparticles at pH 5.5.

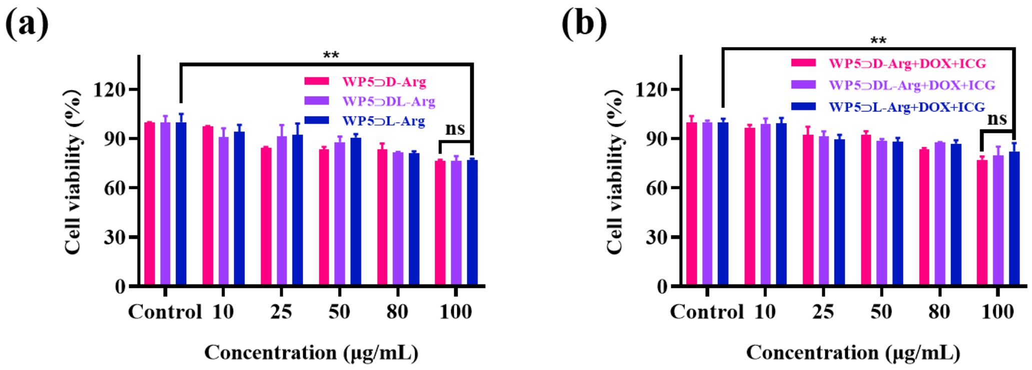

Fig. S22. (a) Relative viability of L02 cell after being incubated with various concentrations of WP5⊃D/DL/L-Arg. (b) Relative viability of L02 cell after being incubated with various concentrations of WP5⊃D/DL/L-Arg+DOX+ICG. *p*-value: ** $p < 0.01$, n.s: no significance.

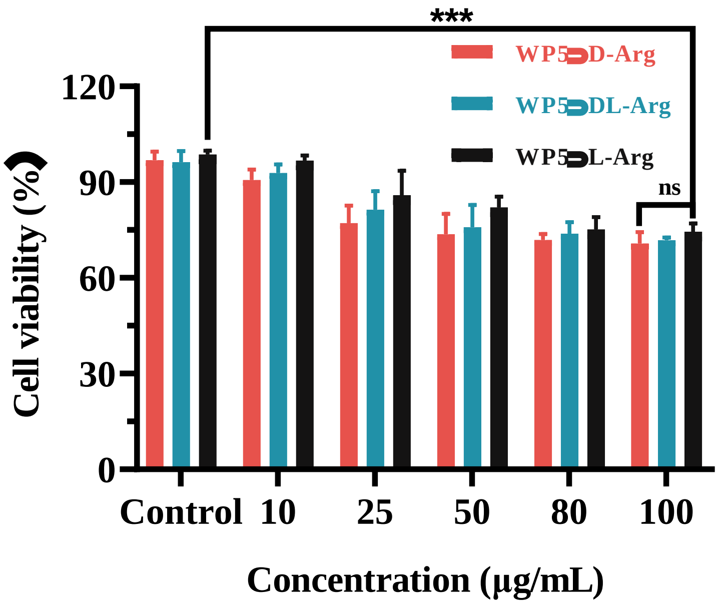

Fig. S23. Relative viability of CT26 cell after being incubated with various concentrations of WP5⊃D/DL/L-Arg. *p*-value: *** $p < 0.001$, n.s: no significance.

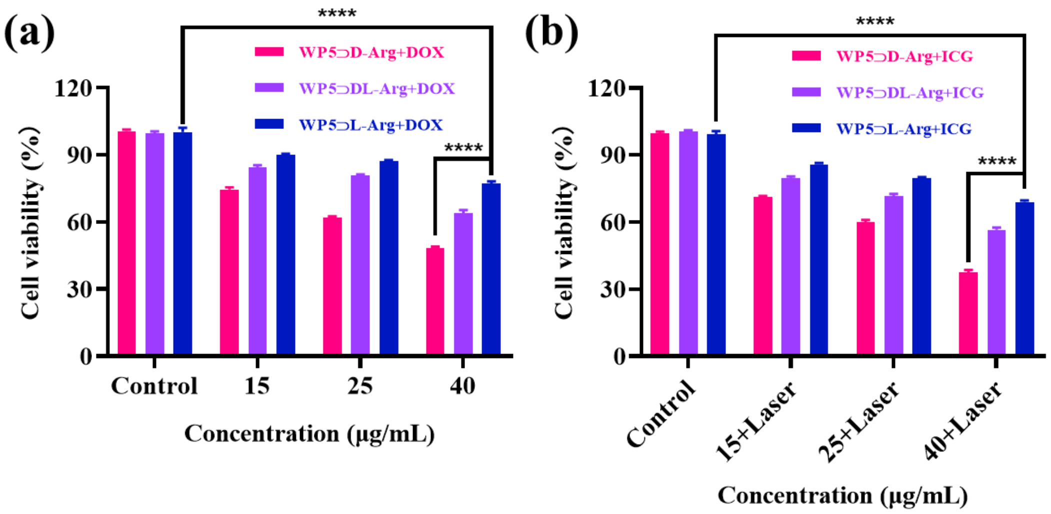

Fig. S24. (a) Relative viability of CT26 cell after being incubated with various concentrations of WP5⊃D/DL/L-Arg+DOX. (b) Relative viability of CT26 cell after being incubated with various concentrations of WP5⊃D/DL/L-Arg+ICG+Laser. *p*-value: **** $p < 0.0001$.

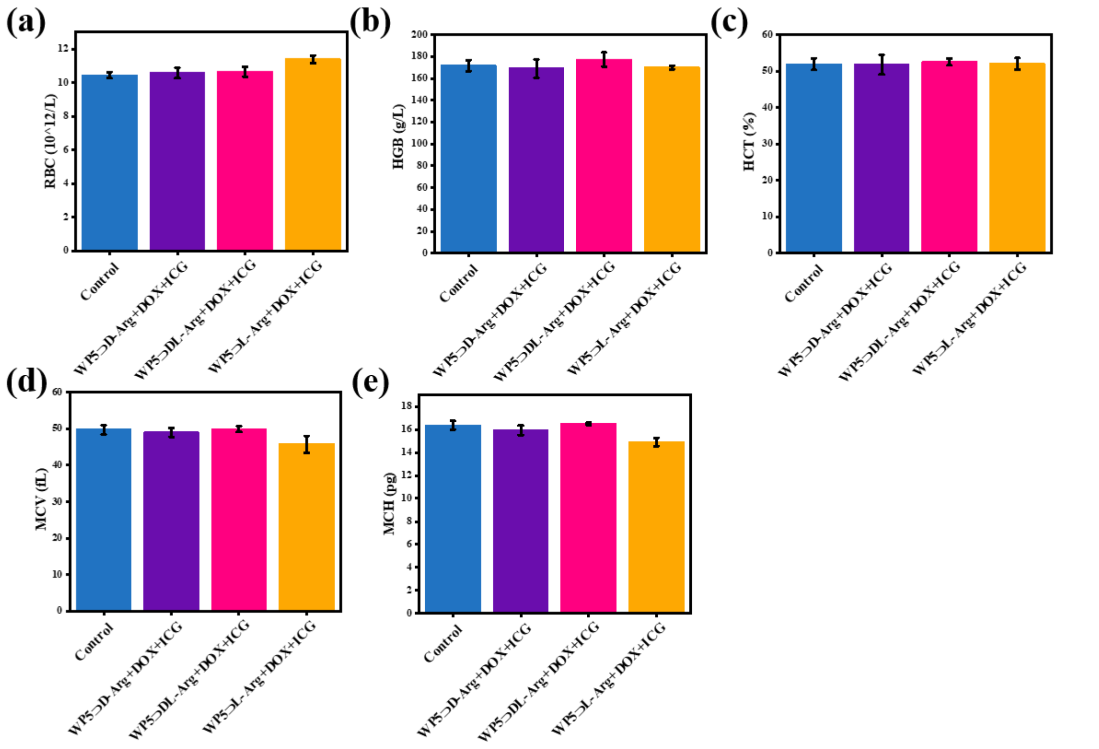

Fig. S25. Blood routine data obtained through i.v. treatment with WP5⊃D/DL/L-Arg+DOX+ICG on day 14, including (a) RBC, (b) HGB, (c) HCT, (d) MCV and (e) MCH. Data were presented as mean ± SD (n = 3).

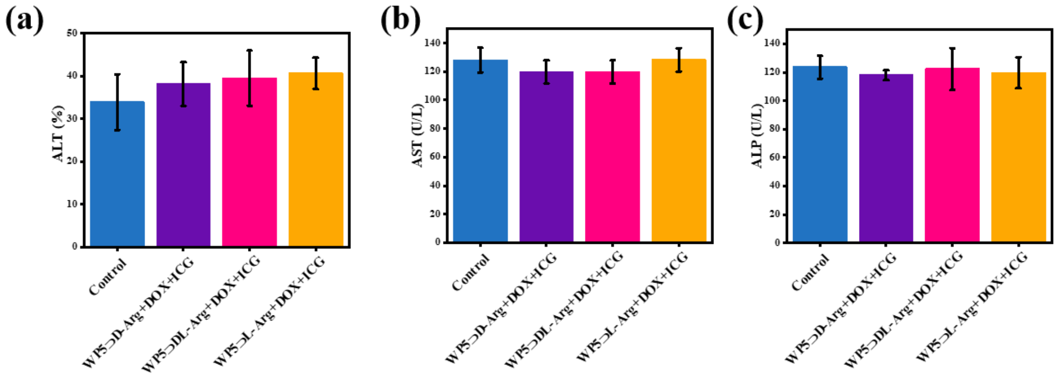

Fig. S26. Serum biochemistry data obtained through i.v. treatment with WP5⊃D/DL/L-Arg+DOX+ICG on day 14, including (a) ALP, (b) AST, and (c) ALT. Data were presented as mean ± SD (n = 3).

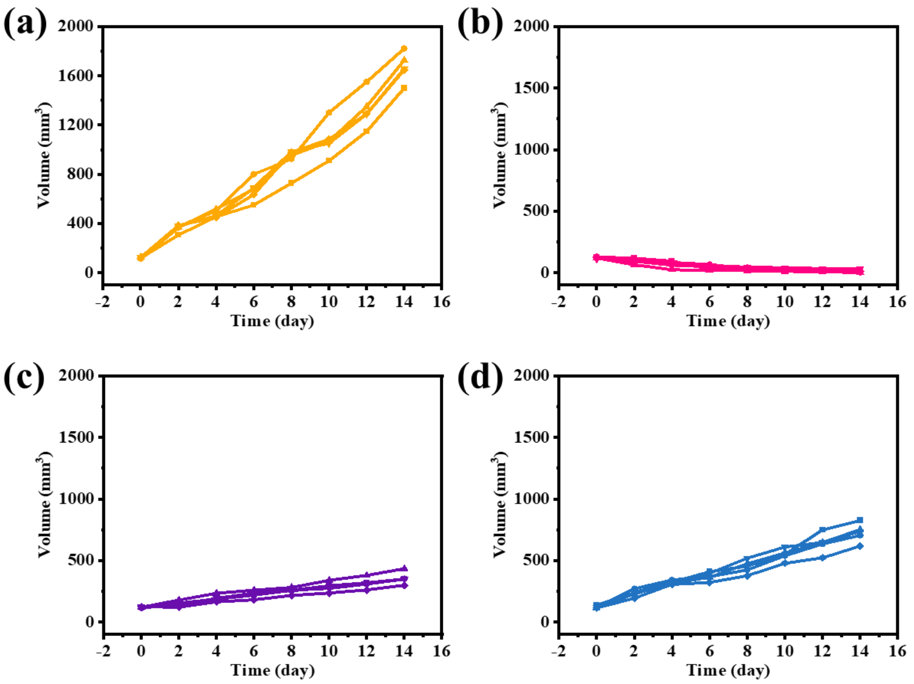

Fig. S27. Tumor growth curves of five mice from (a) control group, (b) WP5⊃D-Arg+DOX+ICG treated group, (c) WP5⊃DL-Arg+DOX+ICG treated group and (d) WP5⊃L-Arg+DOX+ICG treated group.

Reference

S1. Li, H. *et al.* Viologen mediated assembly of and sensing with carboxylatopillar[5]arene-modified gold nanoparticles, *J. Am. Chem. Soc.* 2013, **135**, 1570-1576.
